# NGSNGS: next-generation simulator for next-generation sequencing data

**DOI:** 10.1093/bioinformatics/btad041

**Published:** 2023-01-20

**Authors:** Rasmus Amund Henriksen, Lei Zhao, Thorfinn Sand Korneliussen

**Affiliations:** Lundbeck Foundation GeoGenetics Centre, Globe Institute, University of Copenhagen, 1350 Copenhagen K, Denmark; Lundbeck Foundation GeoGenetics Centre, Globe Institute, University of Copenhagen, 1350 Copenhagen K, Denmark; Lundbeck Foundation GeoGenetics Centre, Globe Institute, University of Copenhagen, 1350 Copenhagen K, Denmark

## Abstract

**Summary:**

With the rapid expansion of the capabilities of the DNA sequencers throughout the different sequencing generations, the quantity of generated data has likewise increased. This evolution has also led to new bioinformatical methods, for which *in silico* data have become crucial when verifying the accuracy of a model or the robustness of a genomic analysis pipeline. Here, we present a multithreaded next-generation simulator for next-generation sequencing data (NGSNGS), which simulates reads faster than currently available methods and programs. NGSNGS can simulate reads with platform-specific characteristics based on nucleotide quality score profiles as well as including a post-mortem damage model which is relevant for simulating ancient DNA. The simulated sequences are sampled (with replacement) from a reference DNA genome, which can represent a haploid genome, polyploid assemblies or even population haplotypes and allows the user to simulate known variable sites directly. The program is implemented in a multithreading framework and is factors faster than currently available tools while extending their feature set and possible output formats.

**Availability and implementation:**

The method and associated programs are released as open-source software, code and user manual are available at https://github.com/RAHenriksen/NGSNGS.

**Supplementary information:**

Supplementary data are available at *Bioinformatics* online.

## 1 Introduction

Since the emergence of next-generation sequencing (NGS), the amount of raw sequencing data generated has expanded rapidly (Hu *et al.*, 2021; Orlando *et al.*, 2021). High throughput sequencing is essentially a massively parallel sampling with a replacement process where we obtain millions or maybe billions of short DNA fragments. These are fragments from specific loci from either parental chromosomes in a diploid genome, the fragments are not direct copies but might be modified due to platform-specific sequencing errors or even possible cytosine deamination which is a hallmark of ancient DNA (aDNA). To analyse the increasing amount of data, several intricate bioinformatical pipelines using multiple tools have become necessary. One essential aspect of genomic analyses is the use of simulated datasets, as a way to assess the performance, validity and reliability of tools during the development of a complex pipeline. This is especially relevant for studies within archaeogenetics, as analyses of aDNA remain troublesome (Prüfer *et al.*, 2010): Chemical and environmental will cause reads to be short and fragmented but will also lead to cytosine deamination as a result of hydrolysis of its amine group (Dabney *et al.*, 2013). Finally, the endogenous levels of aDNA are often low and plagued by contamination that can occur at any point from deposition to sequencing, including bacteria and present-day humans (Willerslev and Cooper, 2005).

In this article, we present a multithreaded next-generation simulator for next-generation sequencing data (NGSNGS) capable of simulating both modern and ancient reads. Our presented tool can simulate reads with similar features as those obtained using already existing simulation tools such as ART (Huang *et al.*, 2012) and gargammel (Renaud *et al.*, 2017).

## 2 Materials and methods

NGSNGS is capable of simulating ancient and modern DNA data as both single end (SE) or paired end (PE), storing the reads in multiple different output formats, including .fasta, .fastq and Sequence Alignment/Map formats (.bam,.sam,.cram). NGSNGS can in its initial step incorporate genetic variations (supplied by a vcf file) to emulate possible parental haplotypes, which followed by the inclusion of the age-associated deamination patterns and read length distributions, makes in-depth analyses of the performance of aDNA pipelines possible. During simulation, NGSNGS can apply additional nucleotide alteration models to the simulated reads to alter the sequence. These include sequencing errors, nucleotide substitutions from a provided (position specific) matrix and stochastic structural variation.

The user can provide adapter sequences and sequencing quality profiles which contain the frequency of quality scores for each nucleotide dependent on its position (cycle) within the read. This is done using the Alias method with constant O(n) preprocessing time and O(1) sampling time. When a nucleotide within the read has been assigned a quality score (with a corresponding error probability) a new base is then sampled with an equal probability between the three remaining nucleotides.

The deamination substitutions can be generated by either the Briggs model (Briggs *et al.*, 2007) or by providing a substitution matrix. The Briggs model parameterizes the signal of post-mortem damage (PMD), using four parameters. One parameter (*ν*) represents the nick frequency, which represents a lack of phosphodiester bonds between adjacent nucleotides for a single strand. The overhang length follows a geometric distribution (*λ*). The deamination rate for the single-stranded overhang part (*δ_s_*) is much higher than the deamination rate in the inner double-stranded part of the fragment (*δ_d_*).

## 3 Results

We assess the correctness of our sequencing error-induced substitutions by comparing the overall cycle-specific mismatch rates between our simulated data and ART. This was done by using various quality scores profiles for ART and NGSNGS, and we obtained identical distributions which were in concordance with our quality scores (see Supplementary Sections 2.2.3 and 4.4.1).

To assess the correctness of our PMD model, we simulated reads with PMD only (no substitutions), for which we tabulated their deamination frequency for both the 5′ and 3′ termini using MapDamage 2.0 (Jónsson *et al.*, 2013). The distribution of the deamination frequency for both the 5′ and 3′ termini showed a symmetrical pattern and was in accordance with expected deamination frequencies calculated by using the probability distribution of the original Briggs model parameters. We found no difference between the expected and observed cycle-specific nucleotide substitutions when simulating sequencing errors or when adding a nucleotide misincorporation file. We identified stochastic insertions and deletions in independent reads. Lastly, we find that we are able to reconstruct using standard genotyping known genetic variants which indicates that the simulated data accurately contains the haplotypes of SNP, insertions and deletions.

### 3.1 Runtime and memory usage

Gargammel is using ART internally for the actual read simulation, thus the overall run time of gargammel will be limited by the run time of ART. We compare the runtimes of NGSNGS, ART and gargammel by using a similar approach to the one used in the ART article (Huang *et al.*, 2012). That is simulating 10^8^ reads from chromosome 17 from the human genome, in a *.fq* format, using the Illumina Hiseq 2500 platform-specific quality scores and assuming a fixed read length 100 base pairs this corresponded to an average depth of coverage of 123.16×. The results are summarized in Table 1 and show that NGSNGS performs faster simulations than both gargammel and ART, regardless of simulating SE and PE. To assess the memory consumption we simulated a larger dataset comprised of 100 million reads from the full GRCh37 human reference genome. NGSNGS used approximately 2.9 gigabytes of memory and the memory usage did not increase as more threads were added. ART in the gargammel pipeline is utilizing the amplicon mode and used 7.9 gigabytes. It should here be noted that the standard reference mode of ART used 1.5 gigabytes.

**Table 1.** The run time is measured by taking the mean wall-clock running time of five repetitions

Program	Running time (s)	
	Single	Paired
NGSNGS w. Sub	613.2	1163.8
ART	1307.2	3026.6
NGSNGS w. Sub + PMD	701.0	1282.6
GARGAMMEL	3791.4	5239.7

*Note*: Each repetition simulates 10^8^ reads, in an uncompressed file format, using a single thread on a server with 88 cores, 754.32 Gb memory. NGSNGS w. Sub, includes sequencing errors with comparable output to that of ART. Finally, NGSNGS w. Sub + PMD includes sequencing errors as well as PMD which makes it comparable to the output of gargammel. Uncompressed output was chosen in NGSNGS to make the comparison similar.

Finally, NGSNGS is developed within a multicore threading framework for which we observe that the runtime decreases as we allocate more cores. Increasing the number of threads with a factor of two decreases the runtime by 40.1%, until reaching 16 threads, from where the decrease rate is reduced (see Supplementary Section 4.1). A relatively large proportion of the runtime of the software is as expected the file writing itself which quickly becomes the bottleneck and the reason why the runtime is not linear in the number of cores allocated.

In conclusion, our implementation and software are faster than current available tested alternatives when using a single thread and get increasingly faster as more threads are allocated while extending the feature set of currently available methods.

## Supplementary Material

btad041_Supplementary_Data
