# Supplementary material for: NGSNGS: next-generation simulator for next-generation sequencing data
Source: Bioinformatics. 2023 Jan 20;39(1):btad041. doi: 10.1093/bioinformatics/btad041 (PMC9891242; doi:10.1093/bioinformatics/btad041)
Supplement: btad041_Supplementary_Data [file btad041_supplementary_data.pdf]

NGSNGS: Next generation simulator for next generation sequencing data

Supplementary Material

Rasmus Amund Henriksen, Lei Zhao, Thorfinn Sand Korneliussen

Contents

1 Introduction **3**

2 Supplementary Method **3**

2.1 Procedure . . . 3
2.2 Random sampling . . . 5
2.2.1 Genome position and Fragment length . . . 5
2.2.2 Sequencing read length and orientation . . . 6
2.2.3 Nucleotide quality and sequencing errors . . . 8
2.2.4 Deamination . . . 8
2.2.5 Misincorporation file . . . 12
2.2.6 Insertion and deletions . . . 13
2.3 Adapter sequences and monophosphate region . . . 14
2.4 Output format specific features . . . 14
2.4.1 Sequence Alignment/Map format . . . 15

3 Supplementary Commands **15**

3.1 NGSNGS with sequencing errors . . . 15
3.2 ART . . . 16
3.3 NGSNGS with deamination . . . 16
3.4 Gargammel . . . 17

4 Supplementary Results **17**

4.1 Time measurements - Wall clock and CPU . . . 17
4.1.1 Wall clock and CPU for uncompressed data . . . 17
4.1.2 Wall clock and CPU for compressed data . . . 20
4.2 Depth of Coverage of simulated sequence reads . . . 22
4.3 Fragment lengths . . . 23
4.4 Sequence alteration models . . . 24
4.4.1 Sequencing error . . . 25

4.4.2 Briggs Deamination model 29
4.4.3 Misincorporation file 32
4.5 Biological variation model 33
4.5.1 Genetic variation - SNP 34
4.5.2 Structural variation - Insertions 35
4.5.3 Structural variation - Deletions 36
4.5.4 Genetic variation - Deamination and Coverage 37
4.6 Stochastic variation model 39

1 Introduction

This document contains the supplementary information for the software NGSNGS version 0.5.0 git commit *cce8263*, it describes the internal design of the software and the test used throughout development. We defer to the Github website for support and run examples, `https://github.com/RAHenriksen/NGSNGS`. NGSNGS is written in C/C++ and has been tested on various platforms, operating system and compilers, including macOS version 12.4, and Linux servers with Red Hat Enterprise release 7.9 and 8.6, htslib is the sole dependencies [1].

The program and method simulate short sequences of DNA similar to the output that is being generated by high-throughput sequencing machines. Data associated with this platform is routinely stored in the fastq format, which in addition to the actual sequence of nucleotides also contains a *sequence identifier* and associates each base with a base quality score which is the error probability which models the platform or machine error which is normally position or cycle dependent, e.g. the quality of sequencing tend to drop along the read.

In this supplementary material we will illustrate the overall procedure of sampling sequence reads, and hereafter describe the three sequence alterations models and two genetic variation models utilized by NGSNGS. The sequence alteration models include simulating platform specific sequencing errors (section 2.2.3), ancient DNA (aDNA) deamination patterns (section 2.2.4) and general nucleotide substitutions from a misincorporation matrix (2.2.5). The genetic variation models include both true biological variation and stochastic structural variations mimicking sequencing error specific variations (section 2.2.6). Following the method description, section 3 will establish the commands used for the simulations when quantifying the time measurements described in the main manuscript. Finally, we will describe the results of multiple analyses performed to substantiate NGSNGS functionality. This includes rigorous analysis of additional time measurements, section 4.1, to further promote our conclusion put forth in the main manuscript, namely that NGSNGS is faster than current available tested alternative next-generation sequence simulators. Additionally we have performed several meticulous analysis to verify that the three sequence alterations models, sequencing error (section 4.4.1), deamination (section 4.4.2) and the general substitutions (section 4.4.3) does incorporate a satisfactory amount of nucleotide substitutions based on a preconceived expectation given information for each of the chosen models. Finally, we perform analysis to show that NGSNGS accurately simulates the true biological variations from individual genotypes (section 4.5) and stochastic structural variations (section 4.6).

2 Supplementary Method

2.1 Procedure

NGSNGS primary sampling procedure is initiated by sampling a desired number of reads (*-r*/*-c*) based on an input fasta file (*-i*), which might contain full human assemblies, scaffolds, contigs, single chromosomes or population haplotypes. For ease of use we have added the functionality to choose subsets at runtime (e.g. *-chr chr3,chr14*). A diagram of the simulation design can be found in Figure 1, illustrating how NGSNGS builds a data structure from the input fasta file containing

the characters of each sequence entry, which can independently be accessed simultaneously by multiple threads to simulate DNA fragments.

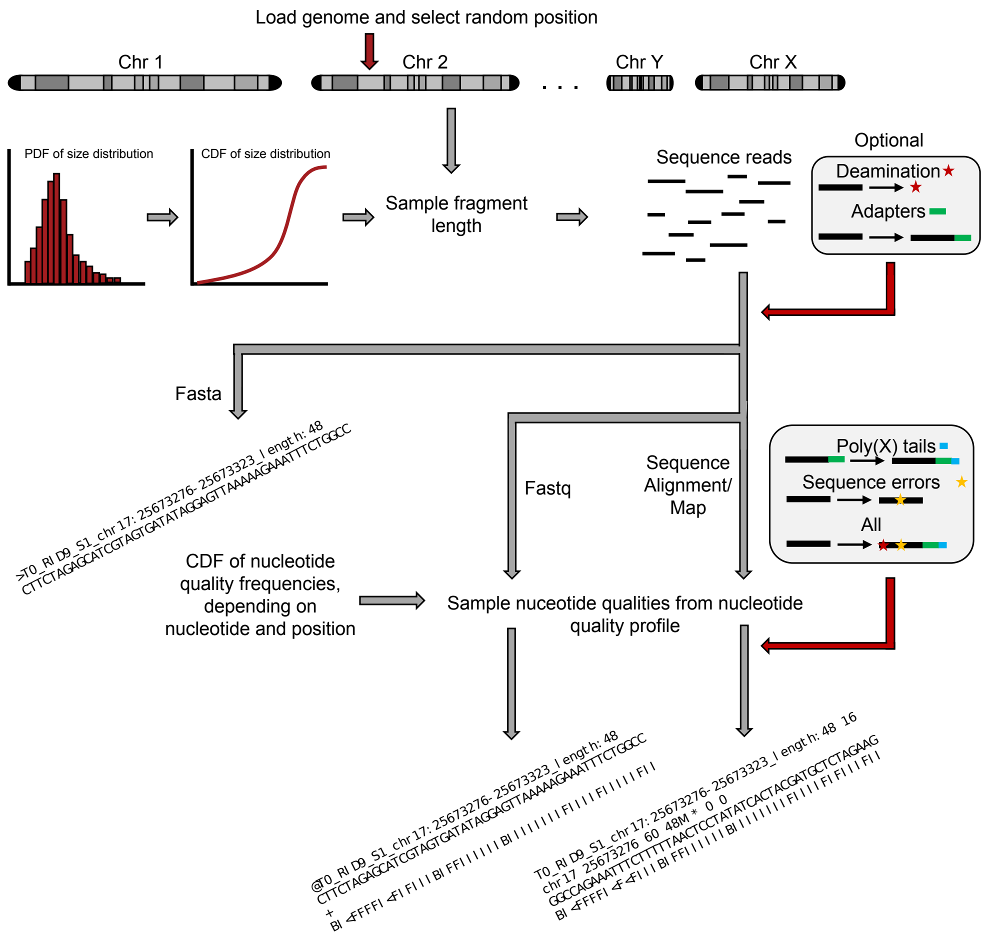

Figure 1: The different steps used by NGSNGS to simulate Next-Generation-Sequencing (NGS) reads, using input files, such as a reference genome, size distribution and quality score profiles. NGSNGS can generate several sequence alterations and store the reads in multiple different output formats, namely fasta, fastq and Sequence Alignment/Map format (SAM) [2].

A secondary approach to the initial step in the simulation procedure is illustrated in Figure 2. If the user supplies a Variant Calling Format (VCF) file (*-vcf* or *-bcf*), the simulation procedure, will sample versions of the desired chromosome (*-chr*) and incorporate the variations representing the haplotypes, emulating the possible multiple parental chromosomes. If the vcf file contains multiple individuals the user can choose which sample to use (selected by *-id*).

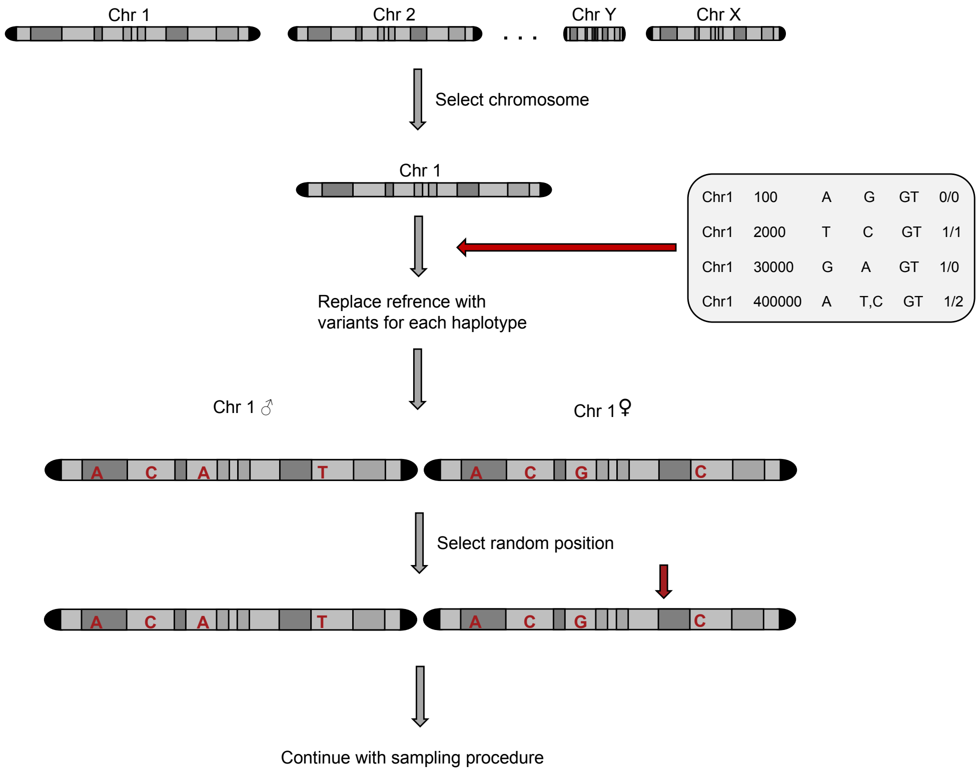

Figure 2: The initialization procedure varies as opposed to Figure 1, by extracting the two haplotypes present in the provided variant calling format file, before incorporating the variations into the chromosomal sequence extracted from the reference genome. Following the replacement of the reference sequence at a specific position given by the vcf, the sampling procedure will proceed as outlined in Figure 1. This visualization is limited to only show SNP variations, with the positions from the illustrated vcf file, solely being visual aid and not the true biological representation of the chromosomal position.

2.2 Random sampling

Throughout the simulation procedure (Figure 1), NGSNGS relies on several pseudo-random numbers, which includes, for the simplest simulation, selecting a chromosomal position, choosing a DNA strand and selection of a fragment length. Depending on the options provided by the user, additional random numbers will be generated when sampling a nucleotide quality score, simulating sequencing errors and deamination amongst other. The random sampling procedures among different threads share a global threadsafe random number generator.

2.2.1 Genome position and Fragment length

The number of reads simulated is determined by either providing the number of reads to be simulated *-r* or desired depth of coverage *-c*. Before generating the sequence reads, we sample a genomic position and determine a fragment length. The genomic position is chosen randomly across the entire genome, with only regions solely consisting of 'N' being discarded. The fragment length is either: 1) of fixed length with option *-l*. 2) Sampled from an empirical length distribution *-lf*. 3) or by choosing one of several either discrete or continuous probability distributions *-ld*.

The length distribution files *-lf* option consists of two columns, the length and the cumulative

frequency, as shown in Table 1.

Length	cumulative Frequency
35	0.00540914
⋮	⋮
190	0.9999406784
191	1

Table 1: Structure of a length distribution file, generated from a subset of the length distribution originating from the Ust-Ishim study [3].

The possible distributions (*-ld*) the user can simulate fragment lengths from, are described in Table 2.

Distribution Name	Input Parameter	Example
Uniform	Uni,Min,Max	Uni,40,180
Normal	Norm,Mean,Variance	Norm,80,30
LogNormal	LogNorm,Mean,Variance	LogNorm,4,1
Poisson	Pois,Rate	Pois,165
Exponential	Exp,Rate	Exp,0.025
Gamma	Gam,Shape,Scale	Gam,20,1

Table 2: The different possible probability density functions the fragment length can be simulated from.

Once a start position and a fragment length has been determined, the sequence of the corresponding fragment is extracted from the reference genome (if variations are to be simulated from the vcf file the possible paternal chromosomes will be generated from the single haploid reference file). This fragment represents a biological molecule that is used as a template sequence for which we generate the sequence reads. To represent both DNA strands, 50% of the generated fragments are reverse complemented, to ensure all fragments comply with a 5' to 3' orientation.

When performing sequencing, the nucleotide content of the organism's genome, might influence specific regions and the number of sequencing reads. As described in article [4], genomic regions with a higher GC content can both have more or less sequencing reads covering that specific region. The articles test, was performed using the illumina sequencing platform of several bacterial genomes, which found depending on the organism the observed GC bias, could both be negative with a lower depth of coverage when compared to the mean or positive with a higher depth of coverage. In the current version of NGSNGS, as previously explained in this section, the genomic position is chosen across the entire genome with equal probability, as such NGSNGS doesn't account for GC bias to simulate either more or fewer reads.

2.2.2 Sequencing read length and orientation

For the simplest output format (*-f .fa* or *.fa.gz*), the generated sequencing reads will be the full representation of the generated fragment, regardless of the fragment length. In this case when

simulating single-end reads, either strand will be represented as illustrated in case A,B in Figure 3, whereas the paired-end reads will represent both DNA strands, facing in opposite direction, with a complete overlap (C,D in Figure 3).

To generate more accurate output files of high-throughput sequencing instruments, when simulating data with the fastq or SAM format, the length of sequence reads depends on the number of sequencing cycles, which can be provided by the *-cl* option. If not provided, the cycle length will be inferred from the dimensions of the sequencing platform specific nucleotide quality profiles (*-q1, -q2*), which represent a quality score distribution for each nucleotide in a given cycle. Throughout this supplementary material with the given commands (section 3), the cycle length is inferred as described in section 2.2.3 to be 150bp. With this approach the sequence read length is independent of the input fragment length parameter, assuming the fragment length is longer than the cycle length.

When the fragment length is greater than the cycle length, the maximum number of bases extracted from the fragment sequence equals that of the sequencing cycle length. In this scenario, for single-end data (E,F from Figure 3) the generated sequencing reads will represent a subsequence of the original fragment sequence, from either strand. Similarly, the paired-end represents two subsequences from both the 5' and 3' end of the fragment sequence (G,H) from both strands facing in the opposite direction, with an inner distance between the end of the first read and the start of the second read. By providing both a fixed fragment length (*-l*) and a cycle length (*-cl*) it will be possible to control the inner distance of the first and second read within the pair, to more accurately represent NGS data of modern samples.

When the fragment length is shorter than the cycle length, as is usually the case for ancient DNA, the simulated sequence reads will be similar to that of case A,B,C,D in Figure 3 with a full representation of the fragment and a complete overlap for paired-end data.

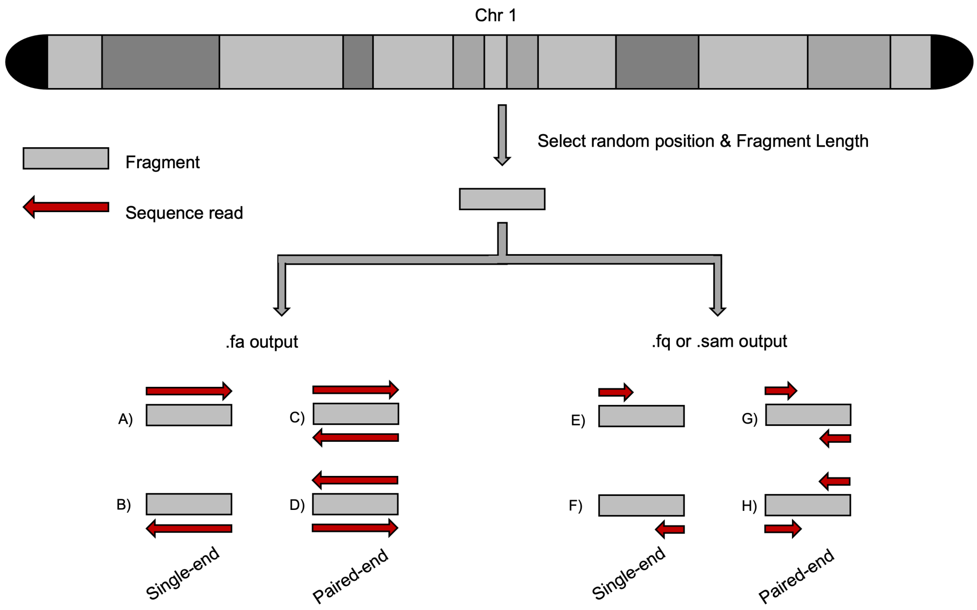

Figure 3: Illustrative representation of the sampling procedure, generating a fragment length from the *-l,lf,ld* option and use this reference genome fragment to simulate sequencing reads, which will differ depending on the chosen output format e.g. *-f fa,fq,sam* and sequencing runs (*-seq SE,PE*).

2.2.3 Nucleotide quality and sequencing errors

Each nucleotide within the sequence has an associated quality score, and each of the quality scores and the raw PHRED scaled quality scores (Q) is logarithmically related to the base-calling error probabilities (p), given by:

$$Q = -10log_{10}(p) \Leftrightarrow p = 10^{-Q/10}$$

Due to the ASCII representation, the quality scores will be binned into a limited number of possible values and are shifted with a value of 33 in fastq and SAM format. In the NGSNGS quality profile format (Table 3), the first row contains the discretized quality scores followed by a row with the base-calling error probabilities. The remaining rows within the profile represent the read cycle with the cumulative distribution function of the quality scores for each position for the nucleotides A, then T, G, C and finally N. The quality strings will be generated by sampling from these cumulative frequencies using the Walker's alias method [5] and converting the corresponding bin quality score (first row within the profile) into an ASCII character. During simulation, the nucleotides will be, given their assigned quality score, altered based on the corresponding incorrect base-calling probability (second row within the profile) to generate an apparent nucleotide substitution by selecting equally between the three remaining nucleotides. If necessary it is possible to simply simulate reads sampled from the reference without any sequencing error alterations by providing the option *-ne*.

Quality	3	7	16	23	28	34	38	41	row 1
Error probability	0.501187	0.199526	0.025119	0.005012	0.001585	0.000398	0.000158	0.000079	row 2
A	5.848056e-07	5.853904e-04	2.188810e-02	2.653555e-02	1.208910e-01	1	0	0	row 3
				⋮					row 4-152
T	6.757693e-07	6.656327e-04	2.267138e-02	2.799307e-02	1.309168e-01	1	0	0	row 153
				⋮					row 154-302
G	2.862653e-07	4.640361e-04	2.050318e-02	2.468781e-02	1.211220e-01	1	0	0	row 303
				⋮					row 304-452
C	1.800824e-07	4.556086e-04	2.281807e-02	2.671451e-02	1.186064e-01	1	0	0	row 453
				⋮					row 454-602
N	1	0	0	0	0	0	0	0	row 603
				⋮					row 604 - 752

Table 3: Structure of the nucleotide quality profile for Illumina Hiseq 2500 sequencing platform with dimensions of 752 rows and 8 columns. To infer the read cycle length it is only the first two rows that are unrelated to the CDF of the position specific nucleotide quality scores, as such the remaining 750 rows contains information for an equal number of positions for the nucleotides A,T,G,C,N. Thus the inferred read cycle length for which the nucleotides can have an associated quality is 150 bp, representing the upper limit of the sequence read length. This profile example is converted from one provided by ART [6].

2.2.4 Deamination

NGSNGS applies two deamination models, representing two different sequencing library preparation used within aDNA studies. The initial model represents a previously described model, with strands ligated to biotinylated adaptors being captured on Streptavidin beads and discarded (referred to throughout this supplementary material as biotin model, *-m b7,nv,Lambda,*$Delta_s$*,*$Delta_d$)

as described in the Briggs model article [7]. The extended model is a *de novo* model developed based on the sequencing library preparation from article [8], which is referred to as the non-biotin model (*-m b,nv,Lambda,Delta$_s$,Delta$_d$*). Both models are utilized to explain the observed post-mortem damage (PMD) pattern i.e., the increased cytosine (C) $\rightarrow$ thymine (T) deamination changes at the lateral parts of the fragments. Such a pattern can be viewed as a joint effect of the formation of 5'-overhangs, the nicks (loss of backbone), and the C to T deamination, along both the single-strand and double-strand parts of ancient DNA fragments. This joined effect can be captured by four parameters: ν which is the chance of a nick occurring per position along each strand, λ for the distribution of the lengths of 5' overhang, δ_d and δ_s is the PMD rate in the double-stranded and single-stranded parts of the fragments respectively. Practically δ_s should be much larger than δ_d, due to the single-stranded region being more unstable compared to the double-stranded region within aDNA, as such the rate of PMD increases.

In the biotin method, only the strand that can not be ligated to the biotinylated adaptors [7] will act as the PCR template, while in our newly developed non-biotin model, both strands of the ancient fragment will contribute to the downstream PCR process. In the non-biotin model, the direction of the adapters attached at both ends determines whether the original strand or its reverse complement serves as the actual PCR template.

The lengths of 5' overhangs of a randomly chosen fragment in both biotin and non-biotin models are assumed to follow a hybrid geometric distribution (with parameter λ) and the Kronecker delta distribution located around length 0. The total length of the left 5' overhang (l) and the right 5' overhang (r) can not exceed $L-2$, where L is the length of the focal DNA fragment. The joint distribution of the lengths of the left and right 5' overhangs of a specific fragment is as follows.

$$\mathbf{P}\left(l, r \,|\lambda, L\right) = \frac{\left[\frac{1}{2}\lambda\left(1-\lambda\right)^{l} + \frac{1_{l=0}}{2}\right]\left[\frac{1}{2}\lambda\left(1-\lambda\right)^{r} + \frac{1_{r=0}}{2}\right]}{S}, \tag{1}$$

where $S = \sum_{l+r\leq L-2}\left[\frac{1}{2}\lambda\left(1-\lambda\right)^{l} + \frac{1_{l=0}}{2}\right]\left[\frac{1}{2}\lambda\left(1-\lambda\right)^{r} + \frac{1_{r=0}}{2}\right]$, and it is a normalising factor. The $1_{l=0}$ and $1_{r=0}$ serve as the corresponding indicator functions.

We use the same version of nick placement formula as in Briggs et al. [7] for the biotin model, described as follows.

$$\mathrm{P}\left(n_{ds} \,|\nu, L_{ds}\right) = \begin{cases} \frac{\nu}{(L_{ds}-1)\nu+(1-\nu)}, & 1 \leq n_{ds} < L_{ds}, \\ \frac{1-\nu}{(L_{ds}-1)\nu+(1-\nu)}, & n_{ds} = L_{ds}, \end{cases} \tag{2}$$

As argued in the biotin model (Briggs et al. [7]), only the nick occurring in the double-strand part will affect the downstream deamination signals, so equation 2 calculates the corresponding conditional probabilities given that one or less nick occurs at double-strand region. n_{ds} represents the nick position in the double-strand part of the focal strand in the fragment (a nick occurs at position n_{ds} means that nick takes place between n_{ds}'th and $(n_{ds}+1)$'th nucleotides in the double-strand part of the focal strand counting from its 5' end). $L_{ds} = L - l - r$ is the length of the double-strand part of the focal fragment. The simulation idea can be summarized as Algorithm 1.

While in the non-biotin model, since both strands of the focal fragment will contribute to the downstream PCR process, we should consider the nicks on both strands. Similarly as argued in the Briggs et al. [7], we assume at most one nick per strand, and only the nicks whose 5' upstream has no other nicks on both strands will be considered (Figure 4). Equation 2 is utilized to simulate the nick position on the first strand n_{ds}, and the following conditional probability, i.e., equation 3, is then applied to simulate the nick position m_{ds} on the second strand (also counting from this strand's 5' end).

$$\mathrm{P}\left(m_{ds}\left|n_{ds},\nu,L_{ds}\right.\right)=\begin{cases}\begin{cases}\nu\left(1-\nu\right)^{m_{ds}+n_{ds}-L_{ds}}, & L_{ds}-n_{ds}\leq m_{ds}<L_{ds}\\ \left(1-\nu\right)^{n_{ds}}, & m_{ds}=L_{ds}\end{cases}, & 1\leq n_{ds}<L_{ds},\\ \begin{cases}\nu\left(1-\nu\right)^{m_{ds}-1}, & 1\leq m_{ds}<L_{ds}\\ \left(1-\nu\right)^{L_{ds}-1}, & m_{ds}=L_{ds}\end{cases}, & n_{ds}=L_{ds},\end{cases} \tag{3}$$

It can be proved the marginal distribution of m_{ds} is identical as that of n_{ds}, i.e., equation 2. A pseudocode of the non-biotin model simulation can be found in Algorithm 2.

Algorithm 1: Simulation Scheme for biotin deamination process

```
Input: An non-deaminated fragment F of the length L, with F_i the i'th nucleotide read;
Output: A deaminated fragment F~;
Initialisation: F~ = F;
Determining the lengths of the left and right 5'-overhangs:
Sampling l and r independently from the hybrid distribution d ~ (1/2)λ(1−λ)^d + 1_{d=0}/2 ;
while l + r > L − 2 do
    Sampling l and r independently from the hybrid distribution d ~ (1/2)λ(1−λ)^d + 1_{d=0}/2;
for position i ← 1 to l do
    if F_i = C then
        if u_1 < δ_s then
            F~_i = T;
for position i ← L − r + 1 to L do
    if F_i = G then
        if u_2 < δ_s then
            F~_i = A;
Determining the position of the nick:
Sampling a nick position n_ds based on Equation 2;
for position i ← l + 1 to l + n_ds do
    if F_i = C then
        if u_3 < δ_d then
            F~_i = T;
for position i ← l + n_ds + 1 to L − r do
    if F_i = G then
        if u_4 < δ_d then
            F~_i = A;
```

Algorithm 2: Simulation Scheme for non-biotin deamination process

Input: An non-deaminated fragment F of the length L, with F_i the i'th nucleotide read, and its reverse complementary strand G;

Output: deaminated strands $\tilde{F}$ and $\tilde{G}$;

Initialisation: $\tilde{F} = F$, $\tilde{G} = G$;

Determining the lengths of the left and right 5'-overhangs:

Sampling l and r independently from the hybrid distribution $d \sim \frac{1}{2}\lambda\,(1-\lambda)^d + \frac{1_{d=0}}{2}$;

while $l + r > L - 2$ **do**

  Sampling l and r independently from the hybrid distribution $d \sim \frac{1}{2}\lambda\,(1-\lambda)^d + \frac{1_{d=0}}{2}$;

for *position* $i \leftarrow 1$ **to** l **do**

  if $F_i = \text{C}$ **then**

    if $u_1 < \delta_s$ **then**

      $\tilde{F}_i = \text{T}$;

      $\tilde{G}_i = \text{A}$;

for *position* $i \leftarrow L - r + 1$ **to** L **do**

  if $G_i = \text{C}$ **then**

    if $u_2 < \delta_s$ **then**

      $\tilde{G}_i = \text{T}$;

      $\tilde{F}_i = \text{A}$;

Determining the position of the nick:

Sampling nick positions on both strands (n_{ds}, m_{ds}) based on Equations 2 and 3;

for *position* $l + 1 \leq i < L - r - m_{ds}$ **do**

  if $F_i = \text{C}$ **then**

    if $u_3 < \delta_d$ **then**

      $\tilde{F}_i = \text{T}$;

      $\tilde{G}_i = \text{A}$;

for *position* $l + n_{ds} < i \leq L - r$ **do**

  if $G_i = \text{C}$ **then**

    if $u_4 < \delta_d$ **then**

      $\tilde{G}_i = \text{T}$;

      $\tilde{F}_i = \text{A}$;

for *position* $L - r - m_{ds} \leq i \leq l + n_{ds}$ **do**

  if $F_i = \text{C}$ **then**

    if $u_5 < \delta_d$ **then**

      $\tilde{F}_i = \text{T}$;

  if $G_i = \text{C}$ **then**

    if $u_5 < \delta_d$ **then**

      $\tilde{G}_i = \text{T}$;

In the above algorithms, u_i ($i = 1, 2, 3, 4$ and 5) are the uniform random variables.

According to both models described above, we can approximate the expected deamination frequency at the first position from the 5' termini, based on the four parameters, i.e., ν, λ, δ_s and δ_d as the following equations,

$$P_d \triangleq \frac{1}{2} + \frac{\lambda}{2}, \quad (4)$$

$$P(\mathrm{C} \to \mathrm{T}|\mathrm{pos.}1, 5') \approx P_d\delta_d + (1 - P_d)\delta_s. \quad (5)$$

We used these theoretical approximations by comparing them to the observed simulated frequencies, with simulations using the original Briggs models by applying the default parameters set forth in the Briggs paper [7] (*-m b*7, 0.024, 0.36, 0.68, 0.0097).

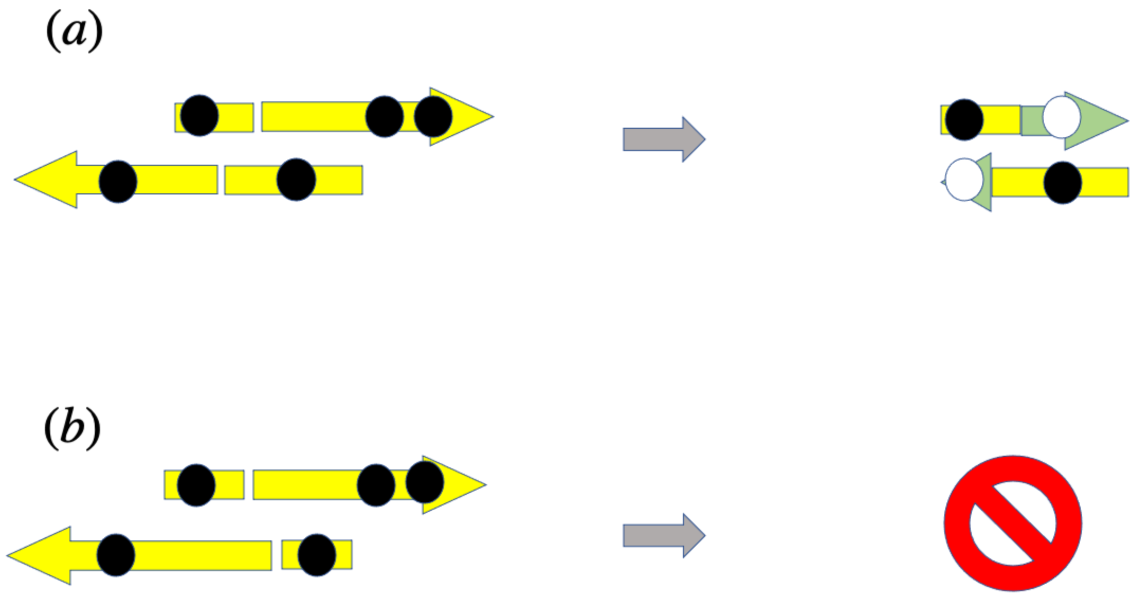

Figure 4: An illustration of nick placement in the model: In both biotin and non-biotin models, only the strands without any nick or the ones with a nick whose 5' upstream has no other nicks on both strands (Case a) will contribute to the downstream PCR process. The strands with at least 2 nicks or those with a nick whose 5' upstream has other nicks on either strand (Case b) are assumed unstable when the adapters are added and will thus be ignored in the model and simulation. In the figure, the black dots along the genome represent the true deamination change, C to T, while the white dots mean the complementary deamination change, G to A.

2.2.5 Misincorporation file

Another substitution model utilized by NGSNGS is the ability to incorporate position specific nucleotide substitution rates from a misincorporation file provided with option *-mf*. This substitution model represent the type specific error probabilities of any of the bases transitioning to any other nucleotide or not transitioning for every cycle or position of the sequence.

Table 4 illustrates a misincorporation with 120 lines, representing substitutions frequencies of each nucleotide for the first and last 15 nucleotides in the 5' and 3' termini respectively.

Termini	To / From	A	T	G	C	row
5'	A	0.865434	0.912348	0.977095	1	1
			⋮			2-15
5'	T	0.011061	0.937133	0.947974	1	16
			⋮			17-30
5'	G	0.043638	0.069117	0.975387	1	31
			⋮			32-45
5'	C	0.011151	0.136731	0.155773	1	46
			⋮			47-60
3'	A	0.913488	0.926578	0.987847	1	61
			⋮			62-75
3'	T	0.059491	0.899341	0.926690	1	76
			⋮			77-90
3'	G	0.157904	0.175423	0.983237	1	91
			⋮			92-100
3'	C	0.036712	0.101059	0.132966	1	101
			⋮			102-120

Table 4: Structure of the misincorporation file, with a substitution pattern for a number of positions from both the 5' and 3' termini of a fragment.

2.2.6 Insertion and deletions

Insertions and deletions (indels) are structural variations (SV) in the genome, that refer to the addition or removal (respectively) of one or more nucleotides. This can either be caused by true biological variations or due to sequencing- or mapping errors. A defining characteristic of the 454 sequencing technology was the platform-specific homopolymer errors, e.g problems distinguishing how many identical nucleotides were observed. For other platforms such as those from Illumina we do not expect homopolymer errors, but rather cycle specific errors as those covered in section 2.2.3. In NGSNGS we can simulate known indels as specified in a vcf file, this will internally generate the parental chromosome(s) and we allow for arbitrary ploidy.

Secondly, we also allow for true random generation of indels, this will, for each fragment serving as the sequence reads template, modify the structural properties of the fragment to mimic indels, i.e. by inserting or deleting nucleotides (*-indel InsProb,DelProb,InsParam,DelParam*). An insertion will cause the fragment to be extended and the added nucleotides will be sampled randomly from the alphabet = "ACGTN", whereas a deletion will shrink the fragment. We here emphasize that this is likely to NOT represent the indel distribution in a population, as we do not model codon usage or frameshift. Here we note that indels are in general poorly understood and the model chosen here is very basic and is likely to be modified in later releases. Based on a user-specified insertion or deletion probability, i.e a Bernoulli random value will be generated to determine whether or not the variation is simulated, and the length of the variations are defined by a geometric distribution given an indel specific parameter.

2.3 Adapter sequences and monophosphate region

When the fragment length is shorter than the sequencing cycle length (see section 2.2.2), concatenation of adapter sequences (*-a1,a2*) at the 3' end of the sequence read extracted from the fragment will be possible. Following concatenation, those sequences still below the sequencing cycle length can have monophosphates appended to the 3' termini as well, until the length is reached. This can either represent poly-A tails or poly-G tails which are a known artifact of NovaSeq sequencing (e.g *-p G*). When simulating the nucleotide qualities, both the sequence read and the adapter sequence will have a quality score assigned, whereas the monophosphate region will have a quality score of 0 assigned.

2.4 Output format specific features

Based on option *-seq SE/PE* the program will either simulate single-end or paired-end data. The format and filetype of the output data is inferred from the filename supplied to the *-f* parameter e.g. *-f fa,fa.gz,fq,fq.gz,sam,bam,cram.* Common for all output formats is the nomenclature of sequence identifier (ID).

```
T<ThreadNumber>_RID<RandomID>_S<Read1StrandInfo>_<Chromosome>:<Start>-<End>
    _length:<Fragmentlength>_<modVal1Val2Val3Val4> F<FragmentNumber> R<
    PairNumber>
e.g.
@T0_RID143_S1_chr1:1498951-1499098_length:148_mod0011 F0 R1
```

The "T<ThreadNumber>" represents which processing thread the read originated from (zero-indexed). "RID <RandomID>" is a randomly assigned value, to distinguish potential reads originating from the same genomic position. "S<Read1StrandInfo>" is a binary value of 0 or 1 signifying the forward- or reverse strand from which the first read is sampled.

The "<chromosome>:<start>-<end>_length:<Fragmentlength>" represents the name of the chromosome (contig/scaffold), chromosomal coordinates and length of the sampled fragment.

The four values stored within <modVal1Val2Val3Val4> represent which of the nucleotide alteration models have altered the sequence of the fragment or sequencing read. The first two are binary values indicating variations of the fragment sequence, namely fragment deamination (*Val1*, section 2.2.4) or alterations based on the misincorporation file (*Val2*, section 2.2.5). The last two indicate variations within the individual reads. *Val3* represents stochastic structural variations, specifically insertions, deletions or both (values *1,2,3* respectively, section 2.2.6). The final value *Val4* is a binary value indicating the simulation of sequencing errors (section 2.2.3).

The value stored within F<FragmentNumber> represents which of the focal fragments, depending on which strand contributes to the downstream PCR process of non-biotin model for deaminated fragments (*-m b,nv,Lambda,*$Delta_s$,$Delta_d$, section 2.2.4). When not simulating data using the non-biotin model the value will be 0 representing the fragment determined by the region extracted from the given strand (S<Read1StrandInfo>).

The final information stored, describes if the sequence read belongs to the first or second read pair, with single-end reads all being annotated as "R1".

2.4.1 Sequence Alignment/Map format

The SAM format (*sam,bam,cram*) usually stores aligned reads in relation to a reference genome, but can also be used as a container for storing the raw sequence data, without alignment information or even storing unaligned reads. We have included the possibility to store the sequence information as well as alignment information.

If the reads are stored to mimic aligned reads, the CIGAR string for the sequencing read itself will be annotated as matches ("M") and soft-clipped ("S") for the adapters and monophosphate region. The mapping quality is set to 60. The value of the FLAG field will be dependent on the sequencing type (single- or paired-end), selected orientation and strand. In this current version the CIGAR string and mapping quality is simply chosen and does not take any of the sequence alteration models into account, so doesn't reflect mismatches, insertions or deletions.

Besides storing the sequence information, the SAM files also contains a header section [2]. In the current NGSNGS version, two tags will be generated, both adhering to the structure defined by the SAM specifications. The first is a reference dictionary "@SQ" tag, comprised of the reference name stored in the "SN" field and the reference genome length in the "LN" field. The second tag is the "@PG" storing program information, the "ID" and "PN" fields are program record identifier and program name, which are identical and equal to "NGSNGS", the "VN" field stores the NGSNGS version, i.e. the git commit hashtag and finally the "CL" field contains the command used for simulating the reads in the given SAM format.

3 Supplementary Commands

To compare the simulation speed across the three tools used throughout this article, we measure the elapsed time of the program starting until it terminates (wall-clock time). To ensure robustness when measuring the wall-clock running time, the measurements in Table 1 in the main manuscript and Table 5 represent the average of five repetitions, which are generated using the commands below. For all tools only a single command is shown, with only the seed values varying between the repetitions ranging from values of one to five. Across all simulation commands in section 3.1 to 3.4, all of the simulated reads in the different output files, have a fixed read length of 100 nucleotides.

3.1 NGSNGS with sequencing errors

The simulation command of NGSNGS from Table 1 (main manuscript) annotated as "NGSNGS w. sub" generates fastq files with platform specific sequencing errors, and as such is comparable with the output from ART (section 3.2). Simulating both single-end (*-seq* SE) and paired-end (*-seq* PE) with seed of 1 (*-s* 1) and a fixed fragment length of a 100 nucleotides (*-l 100*). As described in section 2.2.2 this NGSNGS command simulates fragments with a fixed length below the inferred cycle length (inferred to be 150, see Table 3), resulting in the simulated reads having a fixed read length being the full representation of the fragment (case A,B,C,D in Figure 3).

```
./ngsngs -i chr17.fa -r 100000000 -s 1 -t 1 -l 100 -seq SE -f fq -q1
    Test_Examples/AccFreqL150R1.txt -o Output_se_e_s1
```

```
./ngsngs -i chr17.fa -r 100000000 -s 1 -t 1 -l 100 -seq PE -f fq -q1
    Test_Examples/AccFreqL150R1.txt -q2 Test_Examples/AccFreqL150R2.txt -o
    Output_pe_e_s2
```

In the command above we are using a nucleotide quality profile converted from the ART commands in the following section.

3.2 ART

ART simulates sequencing data by imitating the sequencing process, including the addition of sequencing errors. Unlike NGSNGS, ART cannot simulate data with a fixed fragment length, however, ART allows for simulated data to have a fixed read length using the (*-l*) parameter (see command below). The ART commands used as a comparison for the wall-clock runtime analyses in Table 1 (main manuscript) mimic the sequencing procedure from Illumina Hiseq 2500 platform.

```
./art_illumina --qprof1 Illumina_profiles/HiSeq2500L150R1filter.txt -i chr17.fa
    -c 100000000 -rs 1 -l 100 -na -o test_se_108_s1

./art_illumina --qprof1 Illumina_profiles/HiSeq2500L150R1filter.txt --qprof2
    Illumina_profiles/HiSeq2500L150R2filter.txt -i chr17.fa -rs 1 -c 100000000 -
    l 100 -p -m 200 -s 0 -na -o test_pe_108_s1
```

We refer to the ART documentation [6] for a further explanation of the parameters utilized in the commands above.

3.3 NGSNGS with deamination

We can simulate SE and PE reads that expands on the previous commands by also incorporating nucleotide substitutions that mimics ancient DNA damage, using the original Briggs parameters (Table 1 from the Briggs paper [7]) (*-m b7,0.024,0.36,0.68,0.0097*, i.e. ν,λ,δ_s,δ_d):

```
./ngsngs -i chr17.fa -r 100000000 -s 1 -t 1 -l 100 -seq SE -f fq -m b7
    ,0.024,0.36,0.68,0.0097 -q1 Test_Examples/AccFreqL150R1.txt -o
    Output_se_e_b_s1

./ngsngs -i chr17.fa -r 100000000 -s 1 -t 1 -l 100 -seq PE -f fq -m b7
    ,0.024,0.36,0.68,0.0097 -q1 Test_Examples/AccFreqL150R1.txt -q2
    Test_Examples/AccFreqL150R2.txt -o Output_pe_e_b_s1
```

Since we're not incorporating adapters in these simulations, the NGSNGS output files with both sequencing errors and deamination will be comparable to the output files generated by gargammels pipeline, by discarding the adapter step of the gargammel.

3.4 Gargammel

We therefore ran the first, second and fourth step, which includes the subprograms "fragsim", "deamSim" and finally parsing the intermediate output files to ART. When performing the time measurement analysis, we ran the three other steps separately and calculated the cumulative time of the three commands.

```
gargammel/src/fragSim -tag e -n 100000000 -m 0 -M 1000 -l 100 chr17.fa >
    simulation.e.fa

gargammel/src/deamSim -damage 0.024,0.36,0.0097,0.68 simulation.e.fa >
    simulation_d.fa
```

These fragmented and deaminated reads can then be parsed to ART, simulating both single- and paired-end reads using amplicon simulation.

```
gargammel/art_src_MountRainier/art_illumina --qprof1 art_src_MountRainier/
    Illumina_profiles/HiSeq2500L150R1filter.txt -na -amp -c 1 -qs 0 -l 100 -i
    simulation_d.fa -o simulation_art_se

gargammel/art_src_MountRainier/art_illumina --qprof1 art_src_MountRainier/
    Illumina_profiles/HiSeq2500L150R1filter.txt --qprof2 art_src_MountRainier/
    Illumina_profiles/HiSeq2500L150R2filter.txt -na -amp -p -c 1 -qs 0 -qs2 0 -l
     100 -i simulation_d.fa -o simulation_art_pe
```

We refer to the Gargammel [9] and ART documentation [6] for a further explanation of the parameters utilized in all of the separate steps described in the commands above.

4 Supplementary Results

4.1 Time measurements - Wall clock and CPU

4.1.1 Wall clock and CPU for uncompressed data

In this initial time measurement, we compare the runtime between ART, gargammel and NGSNGS by investigating the single-threaded performance. In Table 1 from the main manuscript and throughout this section, the measurements are quantified from the simulated data in an uncompressed format (*-f fq*). The measurements include the elapsed wall-clock time and the CPU-time. In Figure 5 the comparable time measurements (NGSNGS w. Error vs ART and NGSNGS w. Error & PMD vs gargammel) are separated by a dotted line. Both Figure 5a and Figure 5b shows, when simulating 10^8 reads, that NGSNGS, within each group with comparable functionality, performs faster with a lower wall-clock and CPU time.

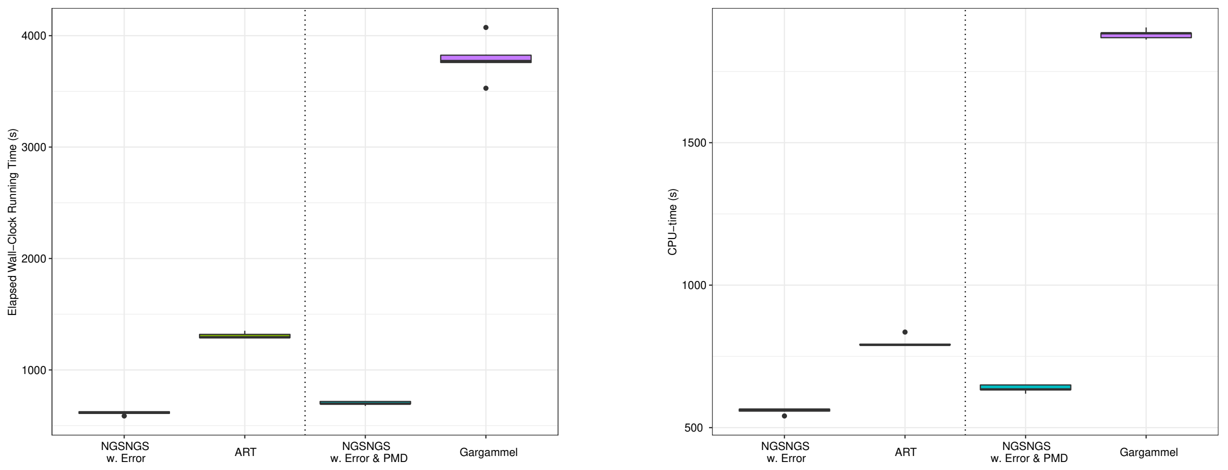

(a) Wall-clock running time

(b) CPU time

Figure 5: Boxplots of values across the five repetitions for each commands used to measure both the wall-clock- and CPU time. The error bars in the boxplot represents the 95% confidence interval, where the bottom and top of the box are the 25th and 75th percentiles, the line within box is the median, and the and any outliers are shown as black colored circles. The group gargammel represent the cumulative time across the three commands: "fragSim","deamSim","ART".

Fair comparison of runtime of programs can be notoriously sensitive on multiuser systems. In Table 5 we show both the mean and the minimum the described scenario showed in Figure 5. We observe that NGSNGS is magnitudes faster in all evaluated metrics, as shown by the speedup metric.

Program	Minimum Running time (s)		Mean Running time (s)	
	Single-end	Paired-end	Single-end	Paired-end
NGSNGS w. Sub (s)	587.00	1144.00	613.20	1163.80
ART (s)	1284.00	2963.87	1307.23	3026.55
Speedup (x)	2.19	2.59	2.13	2.60
NGSNGS w. Sub + PMD (s)	677.00	1259.00	701.00	1282.60
Gargammel (s)	3528.10	4143.672	3791.43	5239.72
Speedup (x)	5.21	3.29	5.41	4.09

Table 5: The minimum and mean running time of the five repetitions for both of the comparable groups (Figure 5), with the speedup when comparing NGSNGS with the other tools.

Since NGSNGS is a multi-threaded application, we also investigated the performance of our software stratifying by number of cores allocated (1,2,4,8,16,32) to the program, with the command using 32 threads (*-t*) shown below. Mean value across 5 repetitions can found in Table 6 when simulating $2 \cdot 10^8$ reads, boxplots of the distributions for the wall-time and CPU-time measurements can be found in Figure 6.

```
./ngsngs -i chr17.fa -r 200000000 -t 32 -s 1 -l 100 -seq SE -q1 Test_Examples/
    AccFreqL150R1.txt -f fq -o output_se_t1_32
```

Threads	1	2	4	8	16	32
NGSNGS w. Sub (single-end) (s)	1253.2	744.4	522.2	392.8	304.2	231.6
Speedup (x)	1	1.68	2.40	3.19	4.12	5.41
NGSNGS w. Sub (paired-end)	2095.00	1191.2	765	515.4	369.8	299.6
Speedup (x)	1	1.76	2.74	4.06	5.67	6.99

Table 6: The mean wall-clock running time of five repetitions with $2 \cdot 10^8$ single-end simulated, reads, and the speedup compared to the wall-clock running time using one thread.

From both Figure 6a and Table 6 we consistently observe faster and faster runtimes as we increase the number of threads (speedup shown in Figure 6c), however it doesn't appear to be a perfect linear relationship. We observe that allocating 32 cores to the program led to a very small increase in CPU time (Figure 6b). In details the overall CPU time goes from the mean value of 1092 (s) to 1187 (s), which is a increase of only 8.70 % (6.88 % increase when comparing the minimum values).

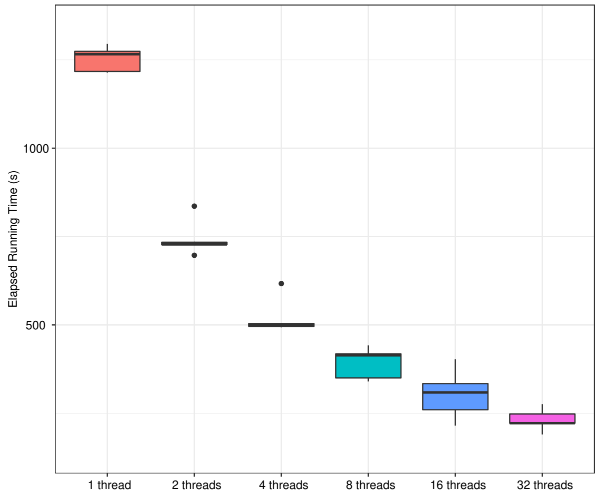

(a) Wall-clock running time

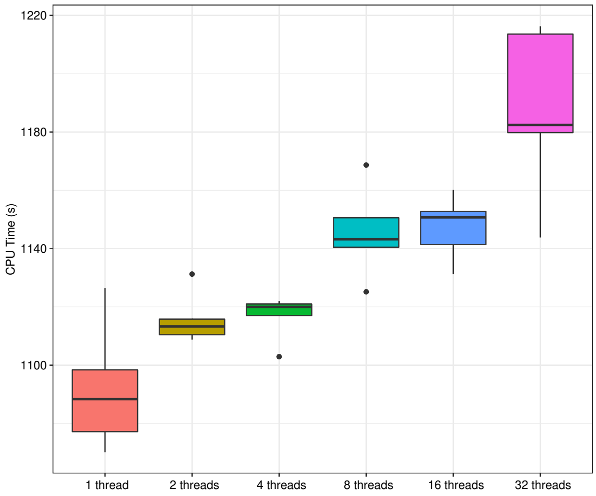

(b) CPU time

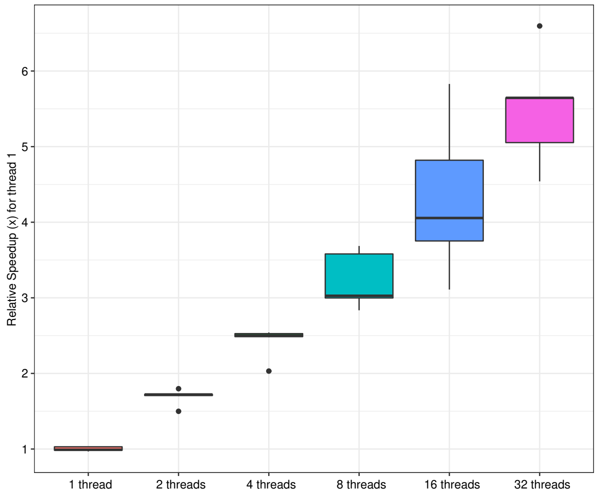

(c) Relative speedup in wall-clock running time

Figure 6: Six different scenarios (NGSNGS w. sub) with 5 replicates in each. We are simulating $2 \cdot 10^8$ reads, outputting these in a fastq file. We are allocating 1,2,4,8,16,32 sampling threads to the program. Notice that the y-axis does not start at zero for Figure 6b.

4.1.2 Wall clock and CPU for compressed data

The threading described in the previous paragraph is the raw sampling computational threads (*-t*). Through the use of sophisticated external libraries (htslib [1]) we also give the user the possibility of writing compressed files using specific compression threads (*-t2*). We therefore replicated the scenario from previous section 4.1.1 but simulating $5 \cdot 10^7$ reads with a fixed number of computational threads to one and now varying the number of compression cores. This analysis is done for both *-f .fq.gz* and *.bam*, see Figure 7 and Figure 8.

```
./ngsngs -i chr17.fa -r 50000000 -t2 32 -s 1 -l 100 -seq SE -q1 Test_Examples/
    AccFreqL150R1.txt -f fq.gz -o output_pe_t2_32
```

```
./ngsngs -i chr17.fa -r 50000000 -t2 32 -s 1 -l 100 -seq SE -q1 Test_Examples/
    AccFreqL150R1.txt -f bam -o output_pe_t2_32
```

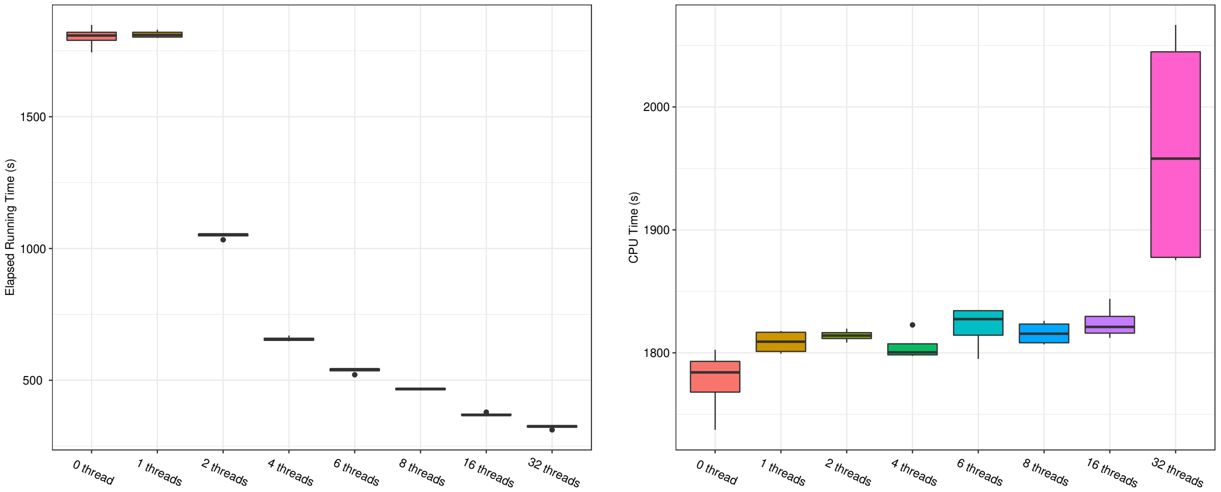

(a) Wall-clock running time

(b) CPU time

Figure 7: Eight different scenarios (NGSNGS w. sub) with 5 replicates in each. We are simulating $5 \cdot 10^7$ reads, outputting these in a fastq.gz file. We are allocating 0,1,2,4,6,8,16,32 compression threads to the program. Notice that the y-axis does not start at zero for Figure 7b.

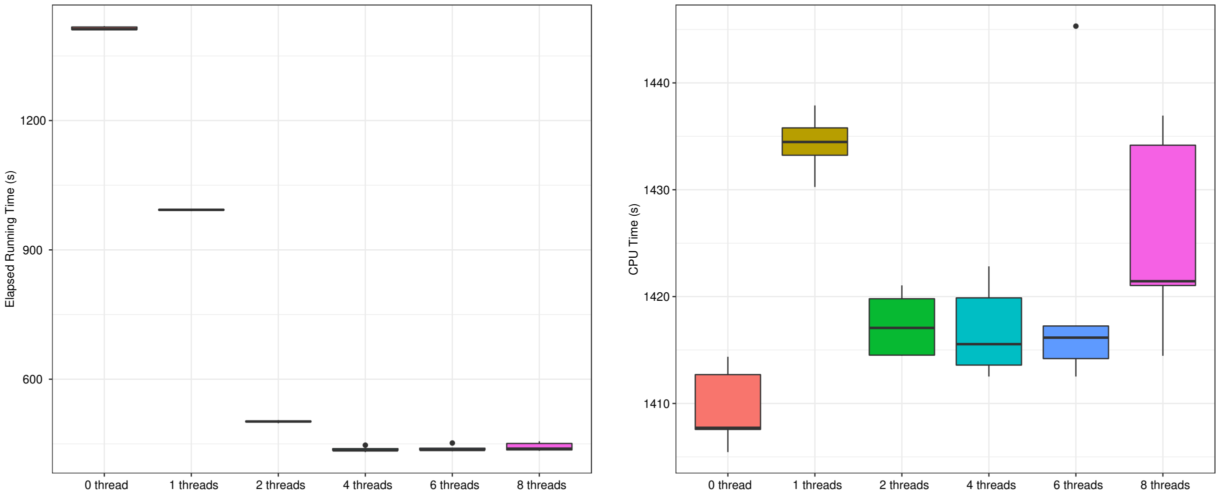

(a) Wall-clock running time

(b) CPU time

Figure 8: Six different scenarios (NGSNGS w. sub) with 5 replicates in each. We are simulating $5 \cdot 10^7$ reads, outputting these in a BAM file. We are allocating 0,1,2,4,6,8 compression threads to the program. Notice that the y-axis does not start at zero for Figure 8b.

As we add more compression cores we observe a decrease in wall-clock, this is similar to the previous analyses where we observed similar trend by increasing the number of computational cores to the program. In Figure 7 the analysis suggests that there would be diminishing returns after around eight cores has been allocated to the program. Interestingly simulating *bam* files in Figure 8 there is no clear benefit from having more than four compression threads in this specific

setup. These analyses suggest the bottleneck in this particular setup becomes the disk IO, the generation of reads or a combination of these factors. It should here be emphasized that this might be very different on another server with faster storage medium or if a different compression level had been specified.

4.2 Depth of Coverage of simulated sequence reads

Initially NGSNGS calculates the mean fragment length from the *-l, -lf, -ld* option and together with the reference genome length (*-i*) NGSNGS will estimate the number of reads required to obtain the desired depth of coverage.

We simulated five repetitions of a different amount of coverage (*-c* 0.05,0.1,0.5,1,5,10,20) of two different fragment length inputs, to ensure this does not affect the desired coverage. In Figure 9 we see the relationship between the desired coverage command (with one example shown below in the command) and the coverage estimated following the alignment of the simulated reads.

```
./ngsngs -i Test_Examples/Mycobacterium_leprae.fa.gz -c 0.05 -t 1 -s 1 -lf
    Test_Examples/Size_dist_sampling.txt -seq SE -q1 Test_Examples/AccFreqL150R1
    .txt -f fq -o Cov005

bwa mem Test_Examples/Mycobacterium_leprae.fa.gz Cov005.fq > Cov005_align.bam

samtools sort Cov005_align.bam -o Cov005_align_sort.bam
samtools depth -a Cov005_align_sort.bam |datamash mean 3
```

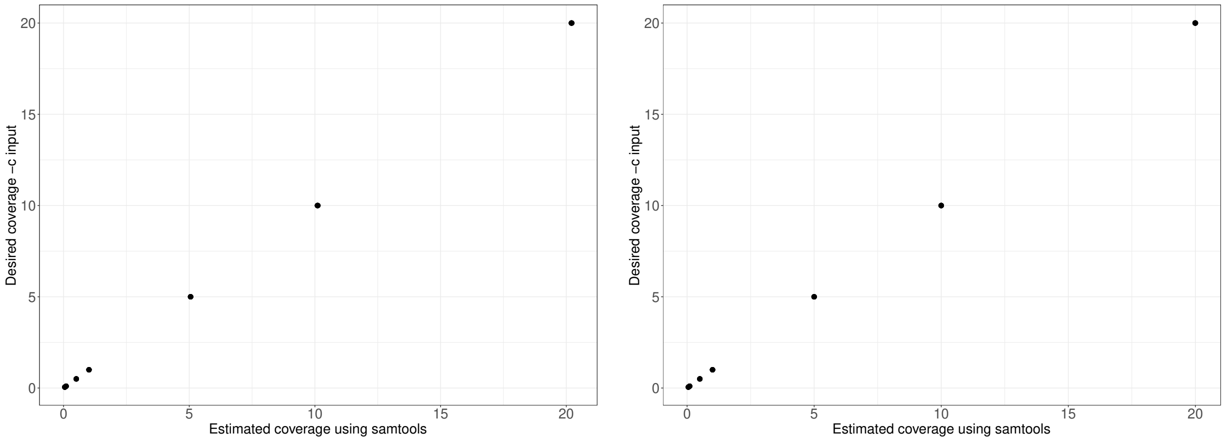

(a) Fragment length *-lf*

(b) Fragment length *-ld Uni,60,140*

Figure 9: Linear relationship between the desired depth of coverage (*-c*) and an estimated depth of coverage of aligned fastq files simulated by NGSNGS. The fastq files was simulated with two different fragment length options, with different distributions.

From Figure 9a and 9b we observe a perfect linear relationship between the estimated and desired depth of coverage. Internally we are using a simple heurestic for obtaining an estimate of the required number of reads for obtaining a desired depth of coverage.

4.3 Fragment lengths

To verify that the different fragment length options (section 2.2.1) also fit the expected distributions, we simulated reads in the fasta format (otherwise the reads would be capped by the cycle length, as explained in section 2.2.1 and 2.2.2), with a single example of the commands shown below.

```
./ngsngs -i Test_Examples/Mycobacterium_leprae.fa.gz -r 100000000 -t 20 -s 1 -ld
    Pois,165 -seq SE -f fa -o MycoBactFaPois
```

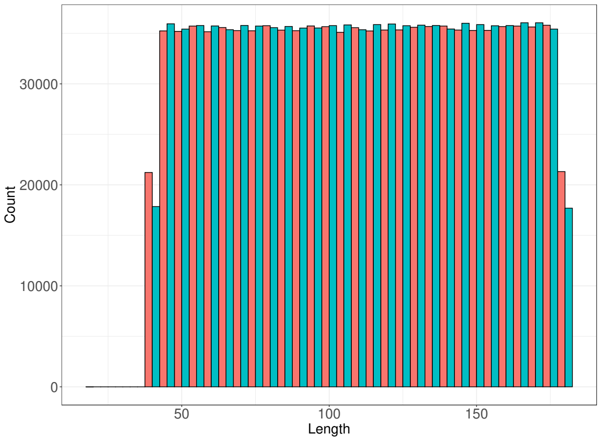

(a) Fragment length from *-ld Uni,40,180*, Uniform distribution in a closed interval $[a,b]$.

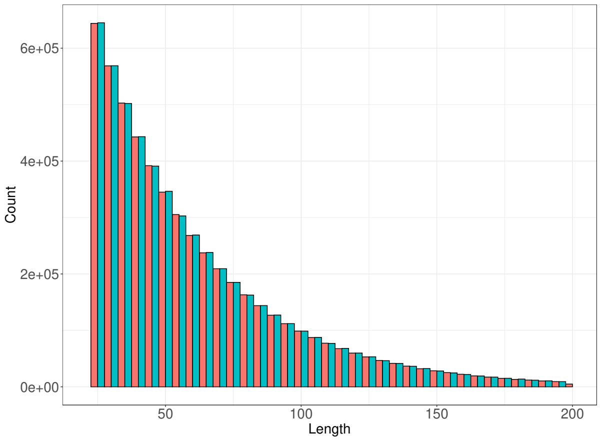

(b) Fragment length from *-ld Exp,0.025*, Exponential distribution with parameter λ.

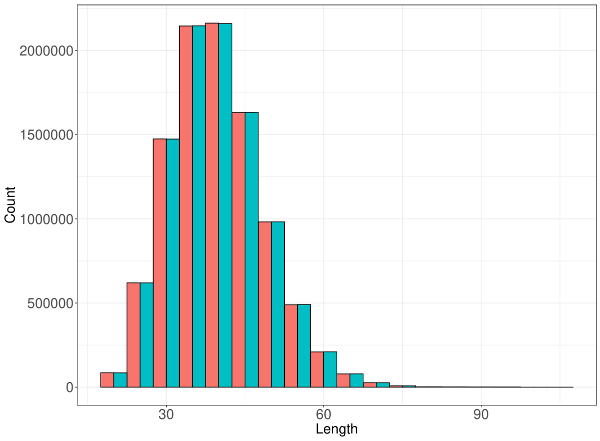

(c) Fragment length from *-ld Gam,20,2*, Gamma distribution parameters α and β.

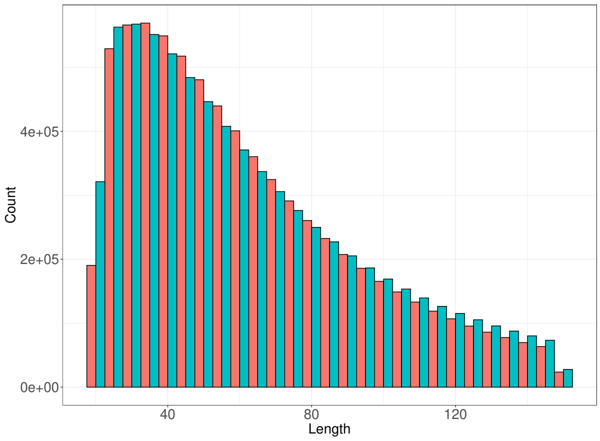

(d) Fragment length from *-ld LogNorm,4,0.8*, Lognormal distribution with the parameters μ and σ.

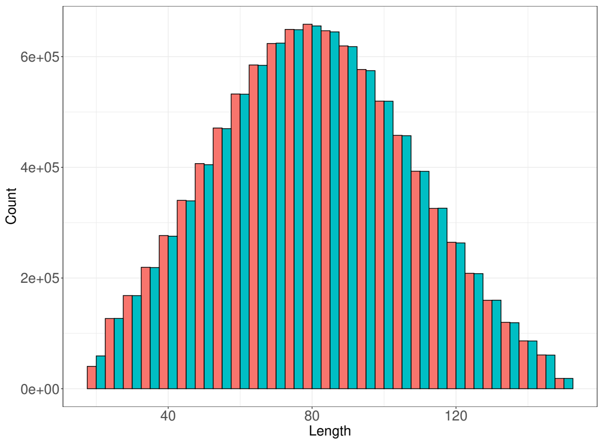

(e) Fragment length from *-ld Norm,80,30*, Normal distribution with the parameters μ and σ.

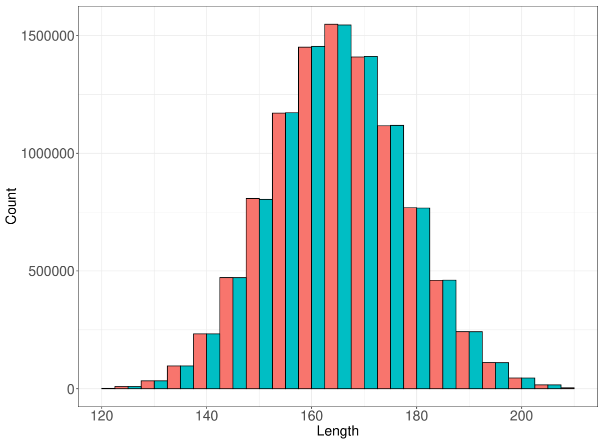

(f) Fragment length from *-ld Pois,165*, Poisson distribution with the parameter λ.

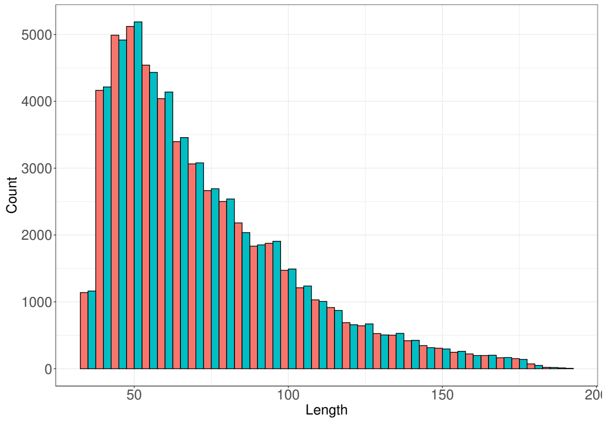

(g) Fragment length from length file
-lf Test_Examples/Size_dist_sampling.txt

Figure 10: Fragment length distributions of the theoretical distributions and simulated NGSNGS reads, with red signifying the teoretical values and blue the simulated NGSNGS data.

Across all the chosen fragment length inputs (*-lf*,*-ld*) we see that NGSNGS simulates fragment lengths in accordance with the expectations (Figure 10). The length distribution follows both the theoretical probability density functions in Figure 10a to 10f and the fragment length file in Figure 10g. This support that the fragment lengths, described in Table 1 and 2, are accurately extracted from the reference genome.

4.4 Sequence alteration models

NGSNGS is capable of generating substitution patterns using platform specific sequencing errors, aDNA characteristics by deaminating the simulated fragments using a Briggs model as well as using the substitution frequencies provided by a misincorporation file to alter the fragment sequence in accordance with a more general substitution pattern.

4.4.1 Sequencing error

The first nucleotide alteration model utilized by NGSNGS is simulating sequencing errors based on the platform specific quality profiles. We constructed a single scenario allowing for a simple and similar test for NGSNGS and ART. All reads is simulated with a fixed length of 100 nucleotides. Our quality scores follows a stairlike distribution (see Figure 11a), it is divided into five regions each corresponding to 20 nucleotides, comprised of a single quality score (Table 7). To accommodate this scenario we generated two artificial quality score profiles, one following the NGSNGS format another following ART's, and then measured the observed substitution frequencies (difference to the reference).

Position in read	1-20	21-40	41-60	61-80	81-100
Quality Score	1	5	10	20	40
ASCII (phred +33)	"	&	+	5	I
Base-calling error probability	0.7943	0.3162	0.1000	0.0100	0.0001

Table 7: The nucleotide qualities and error probabilities for the artificial nucleotide quality profiles.

We expect for the first 20 positions of our read that 0.2057 of the nucleotides are correctly called, which is confirmed by Figure 11a and in the diagonal of Table 8. When focusing on a single region from position 21 to 40, we expect a correct base calling frequency of 0.6838, where we see that the frequencies across all of the position specific nucleotide matches vary from a minimum value of 0.680559 to a maximum of 0.686943, with a mean of 0.6837604. This slight variation across the positions within the region, is a result of the pseudo-random numbers, and all values are still in close proximity of the expected values (Figure 11b). This slight variation is observed for all regions, for both the simulated reads from ART and NGSNGS.

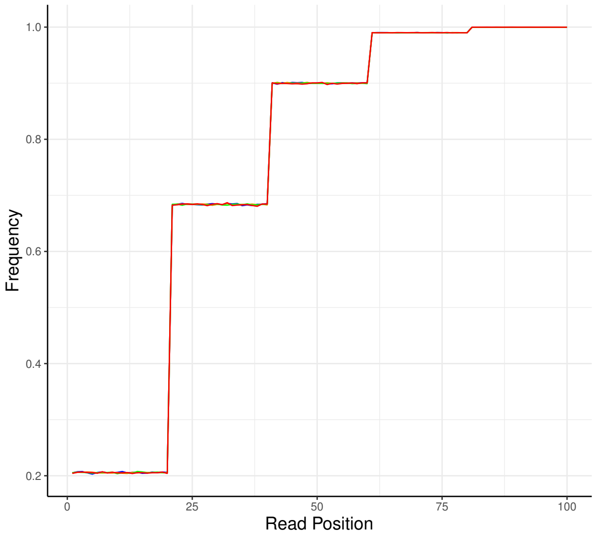

(a) Frequency of correct basecalling across all five regions.

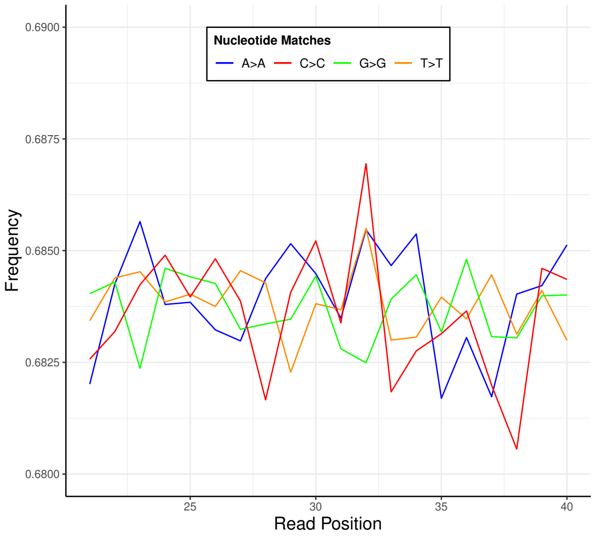

(b) Frequency of correct basecalling across the region ranging from position 21 to 40.

Figure 11: a) The frequency of correctly called nucleotides for 10^6 simulated reads using the artificial nucleotide quality profile for NGSNGS. b) Across position 21 - 40 within the read, each nucleotide have the nucleotide quality score of 5 assigned, with a corresponding correct basecalling probability of 0.6838 % (Table 7).

With a correct base calling frequency of 0.2057 (position 1-20) we expect the error probability of 0.7943 to be uniformly distributed, with a substitution frequency of 0.2647 for the remaining three nucleotides. The observed substitutions simulated by NGSNGS does fit with this expectation as seen in Table 8. In addition the mismatch patterns across all regions are in accordance with this uniform substitutions expectation (Figure 12), albeit with a similar slight variation between the nucleotides in each region as observed for Figure 11b.

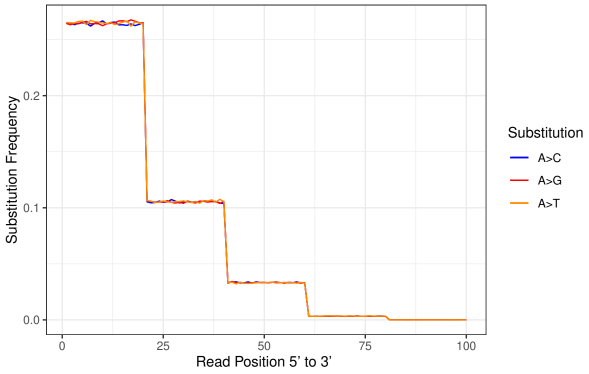

(a) Nucleotide substitution of adenine.

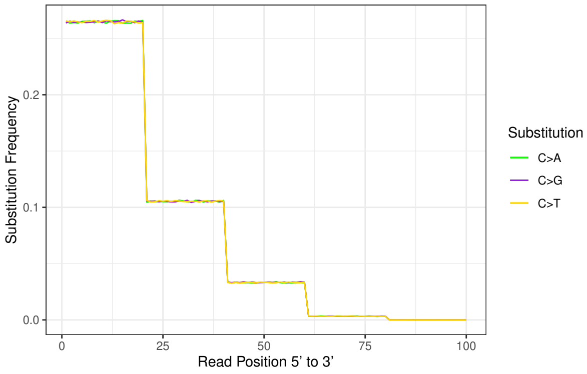

(b) Nucleotide substitution of cytosine.

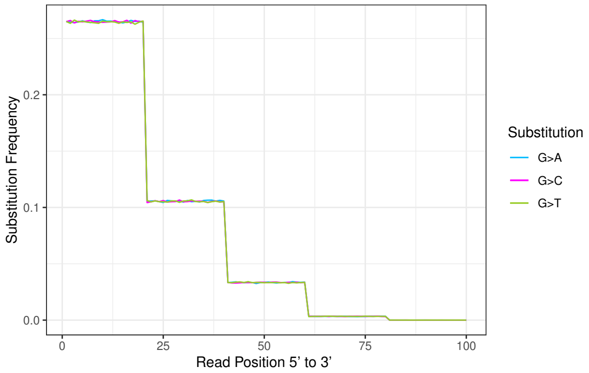

(c) Nucleotide substitution of guanine.

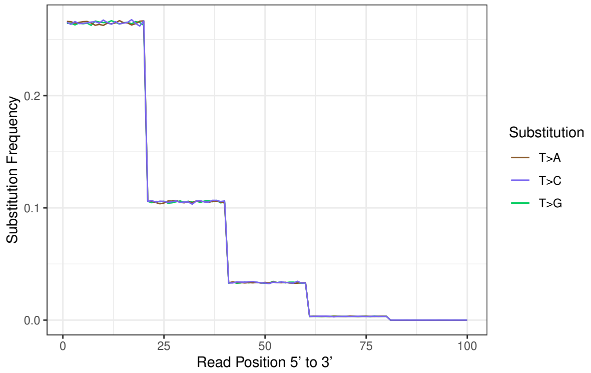

(d) Nucleotide substitution of thymine.

Figure 12: The substitution frequencies representing incorrect base calling for all four nucleotides and their respective substitutions using the NGSNGS quality profile.

From \ To	A	C	G	T
A	0.2057586	0.2642202	0.2649906	0.2650306
C	0.2647405	0.2055446	0.2648324	0.2648825
G	0.2649733	0.2649593	0.2056127	0.2644548
T	0.2647065	0.2648567	0.2650266	0.2054102

Table 8: The average nucleotide substitution frequencies for position 1-20.

The base calling errors for the artificial ART profile (Figure 13) show a similar pattern as NGSNGS (Figure 12), supporting that NGSNGS successfully simulates NGS reads with similar characteristics to those obtained if simulating reads using ART.

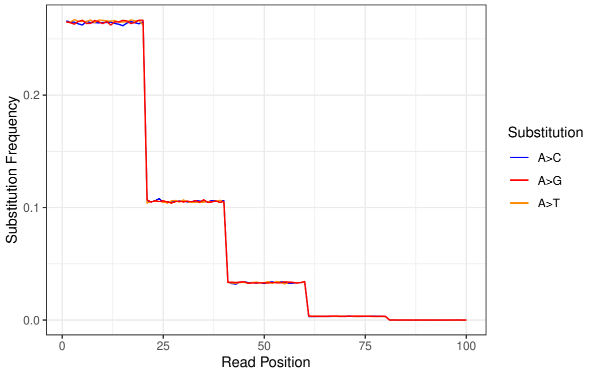

(a) Nucleotide substitution of adenine.

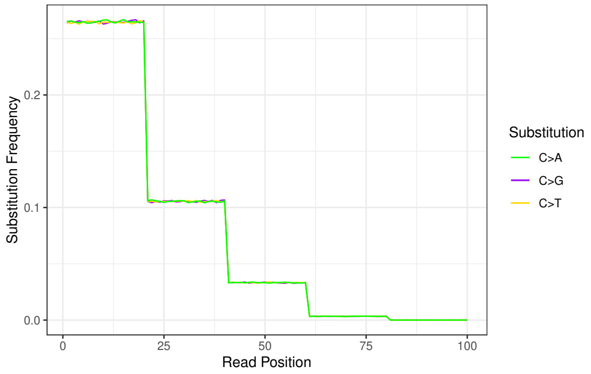

(b) Nucleotide substitution of cytosine.

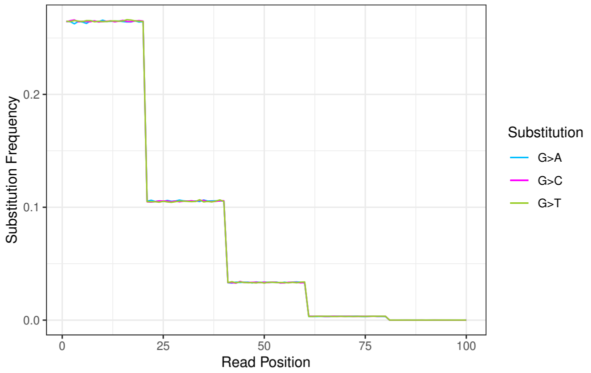

(c) Nucleotide substitution of guanine.

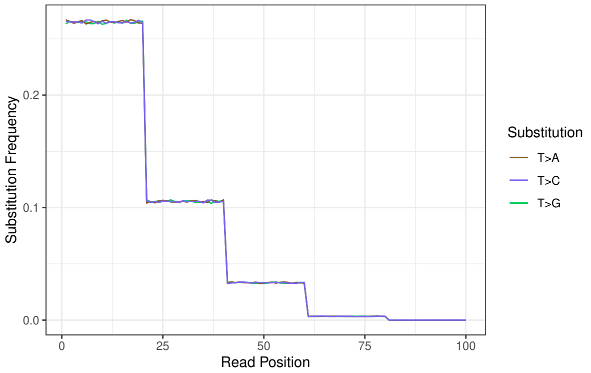

(d) Nucleotide substitution of thymine.

Figure 13: The substitution frequencies representing incorrect basecalling for all four nucleotides and their respective substitutions using the artifical ART quality profile, with similar pattern to those obtained using NGSNGS in Figure 12.

We stratified the simulated reads of NGSNGS by their orientation, results shown in Figure 14 where we observe near identical mirroring of substitution frequencies.

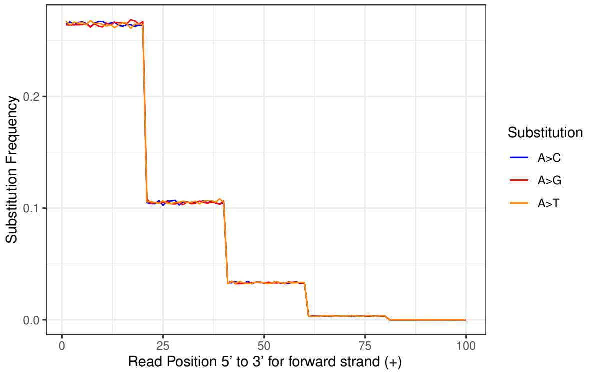

(a) Adenine substitution on forward strand.

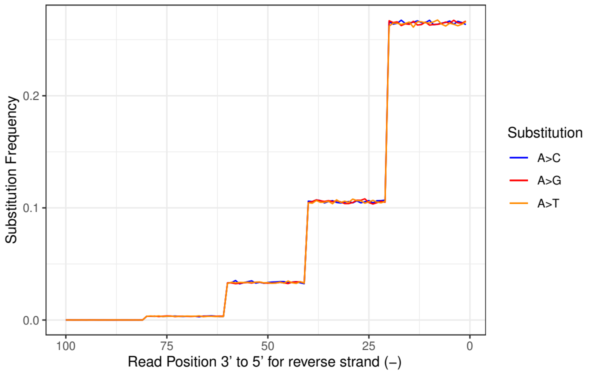

(b) Adenine substitution on reverse strand.

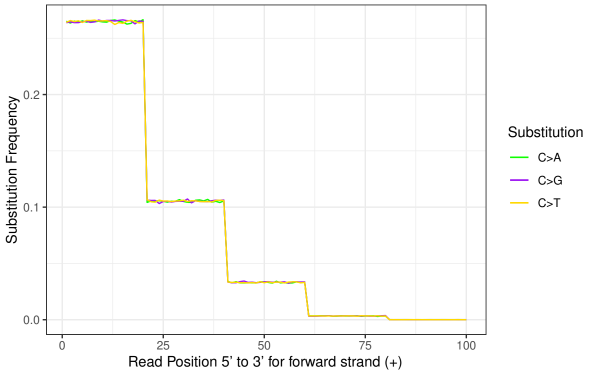

(c) Cytosine substitution on forward strand.

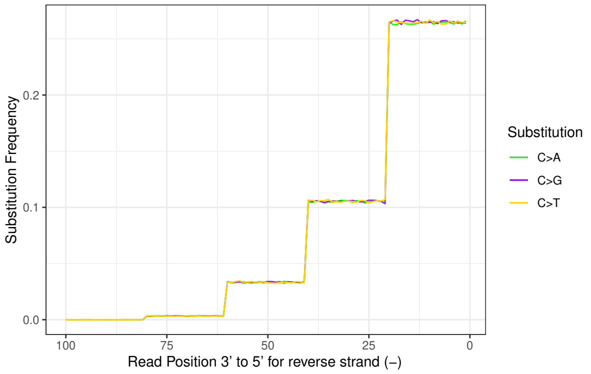

(d) Cytosine substitution on reverse strand.

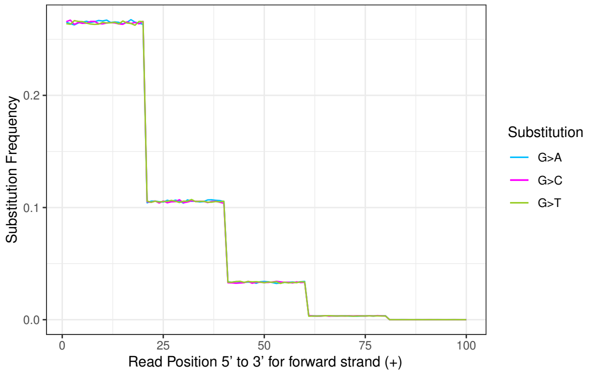

(e) Guanine substitution on forward strand.

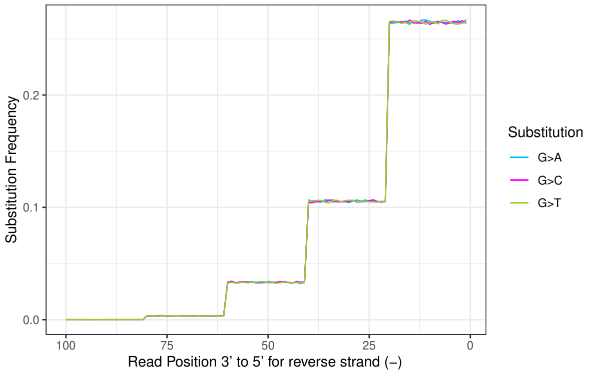

(f) Guanine substitution on reverse strand.

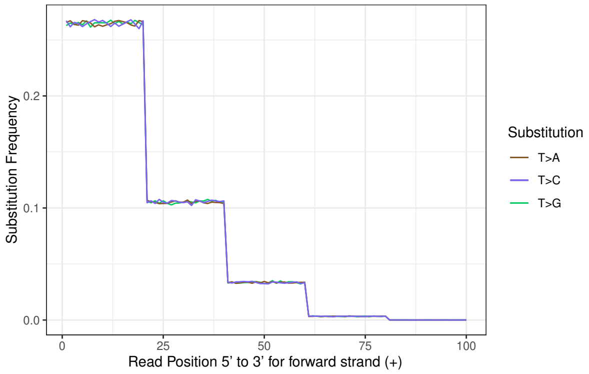

(g) Thymine substitution on forward strand.

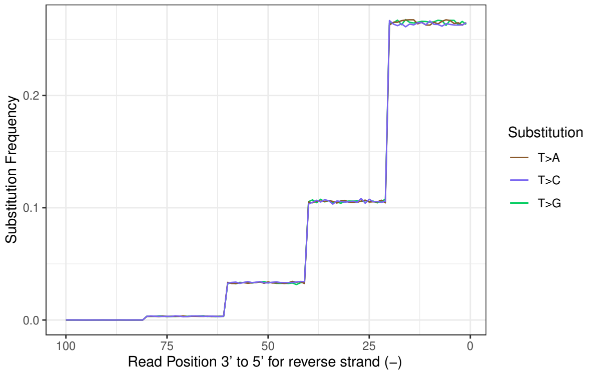

(h) Thymine substitution on reverse strand.

Figure 14: Strand specific nucleotide substitutions using the artifical nucleotide quality profiles.

4.4.2 Briggs Deamination model

When generating cytosine deamination pattern in the 5' and 3' end (respectively) using the Briggs parameters with our biotin model (*-m b7,nv,Lambda,*$Delta_s$*,*$Delta_d$), we expect two requirements to be upheld. Firstly, the deamination pattern should be symmetric in both ends of the fragments, and secondly deamination follows a similar distribution for both the forward and reverse strands. To verify this, we used MapDamage2 [10] to estimate the deamination frequencies for either end. By simulating ten repetitions of 10^6 reads we found that NGSNGS does simulate a deamination pattern in the fragment ends (Figure 15) which is both symmetric and accurate when compared to the misincorporation plot in Figure 3 of the Briggs paper [7].

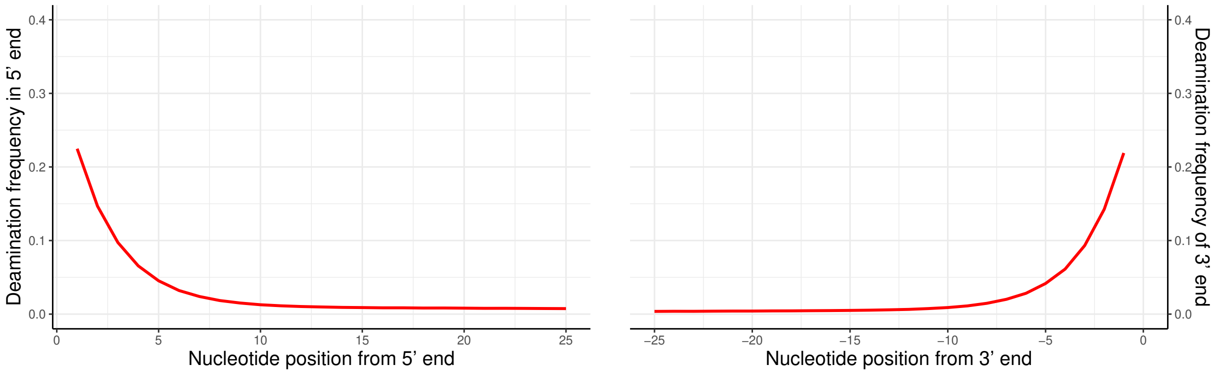

(a) 5' Cytosine to Thymine substitution

(b) 3' Cytosine to Thymine substitution

Figure 15: Deamination frequencies in the first 25 base pairs from the 5' and the last 25 base pairs in the 3' end. The substitution frequencies of C to T at position 1 in the 5' termini can be estimated (using equation 5) to be 0.2241. For both ends the deamination frequencies decrease exponentially over the following 25 base pairs.

Additionally we performed five rounds of 50 repetitions each with 10^6 simulated reads with different seeds. This was done to assess the robustness and accuracy of our deamination model.

To investigate the second requirement, namely that the forward and reverse strand follow the same distribution, we analyse the forward and reverse strand separately. The estimated deamination frequencies across the strands for the five groups of 50 sets are only shown for the first position (Figure 16), with each group being separated by a vertical grey dotted line and the horizontal colored dotted lines representing a theoretical value.

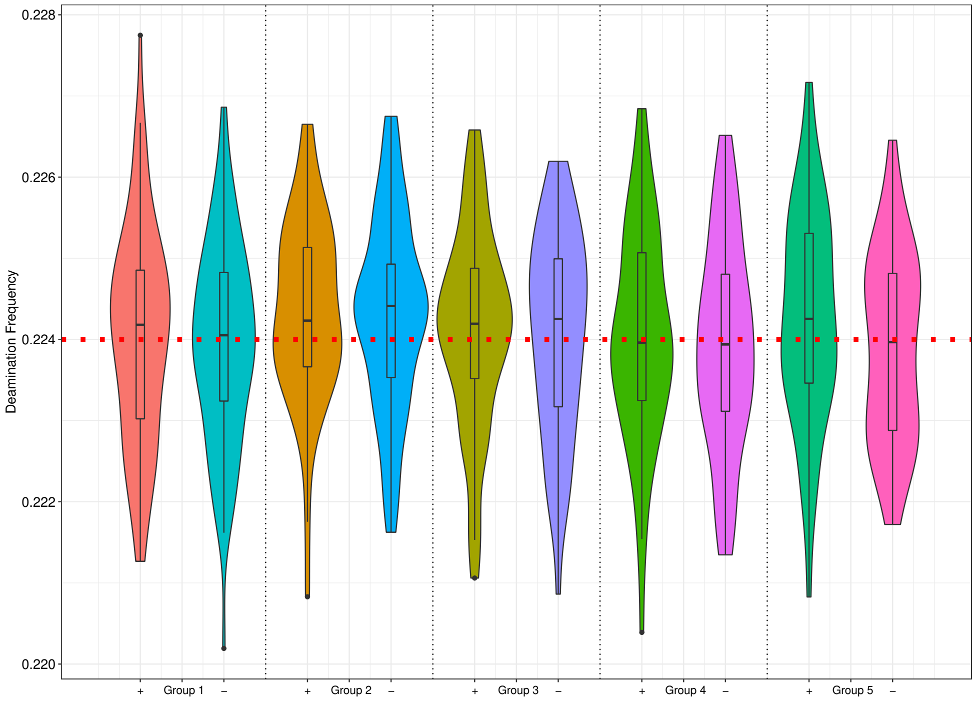

(a) 5' Cytosine to Thymine deamination distribution of five repetitions of 50 bam files separated into forward strand (annotated with "+", flag 0 in the *.bam* file) and reverse strand ("-", flag 16 in the *.bam* file). The red dotted line signifies the expected theoretical substitution frequency for position 1 from 5' end.

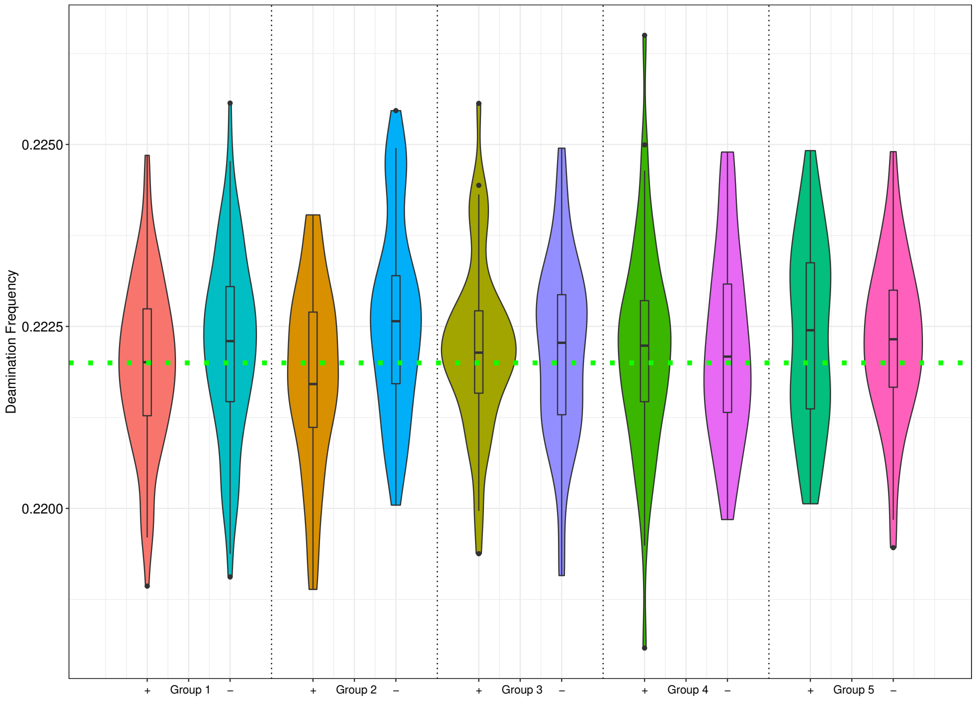

(b) 3' Cytosine to Thymine deamination distribution of five repetitions of 50 bam files separated into forward strand ("+") and reverse strand ("-"). The green dotted line signifies the expected theoretical substitution frequency for position 1 from 3' end.

Figure 16: Frequency distribution of deamination for first position for the deamination changes at the lateral parts of the fragments.

We used the Kolmogorov–Smirnov test between the strand specific deamination distributions to investigate if the deamination patterns uphold the second requirement of following a similar distribution. The Kolmogorov–Smirnov test quantifies a distance between these two empirical distribution functions under the null hypothesis that the samples are drawn from the same distribution, with p-values shown in Table 9.

Deamination	group 1	group 2	group 3	group 4	group 5
C>T in 5' end	0.82765	0.87681	0.96962	0.8768	0.28270
G>A in 3' end	0.24881	0.04239	0.56156	0.96962	0.72801

Table 9: The p-values for each of the Kolmogorov–Smirnov test performed on the different groups in Figure 16a and 16b.

All p-values besides the second group of G to A deamination the 3' end are not significant (Table 9). As such we are unable to reject the null hypothesis for nine out of ten groups, which makes the majority of the deamination frequency patterns consistent across both strands supporting their origin from the same distribution. Thus fulfilling the second requirement as well.

4.4.3 Misincorporation file

The last substitution model, incorporates the sequence modification from a supplied file (*-mf*, Table 4). We expect the simulated mismatch rates to comply with the frequencies in the supplied misincorporation file, and based on 10^8 reads we compared the observed mismatch with the expected values. The expected count of substitutions for position 1 in 5' termini and the quantified values for the simulated reads are seen in Table 10 and show slight variations between the observed and expected count. This difference is an artefact of the sampling process. We calculated the relative error (see Table 11) to quantify that the observed values are compliant with the expected values based on the misincorporation file.

From \ To	A	C	G	T	Sum	
A	23560570.0	623897.0	1764372.0	1275608.0	27224447.0	Observed
	23560962.0	623576.0	1762701.0	1277208.0	27224447.0	Expected
C	253820.0	19325656.0	335617.0	2859585.0	22774678.0	Observed
	253960.4	19325453.0	335220.5	2860044.1	22774678.0	Expected
G	993937.0	560244.0	20638511.0	580536.0	22773228.0	Observed
	993778.1	560517.5	20638693.3	580239.1	22773228.0	Expected
T	300271.0	1415557.0	295496.0	25216323.0	27227647.0	Observed
	301165.0	1416545.6	295174.9	25214761.5	27227647.0	Expected

Table 10: The observed count of mismatches when compared to a reference genome and the expected count of mismatches based on the frequencies of misincorporation file in Table 4.

From \ To	A	C	G	T
A	1.664045e-05	5.148394e-04	9.478237e-04	1.252503e-03
C	5.529774e-04	1.050341e-05	1.182847e-03	1.605091e-04
G	1.598712e-04	4.878720e-04	8.834840e-06	5.117266e-04
T	2.968484e-03	6.978687e-04	1.087758e-03	6.192751e-05

Table 11: The relative error between the observed nucleotide substitution and the expected substitutions when utilizing a misincorporation file (*-mf*).

Given a position i, nucleotide j, $j \in \{A, G, C, T\}$ from the simulated reads and the reference nucleotide k, $k \in \{A, G, C, T\}$ for an equivalent genomic position, then it is possible from our observed values to calculate the expected absolute mismatch values using the misincorporation file (*-mf*, as done for Table 10).

$$N_{ij} = \sum_k O_{ijk} = \sum_k E_{ijk} \tag{6}$$

$$E_{ijk} = N_{ij} \cdot P_{ijk} \tag{7}$$

Where O_{ijk} and E_{ijk} are the numbers of cases and expected number of cases (given N_{ij} the total number of nucleotides at position i whose reference is j is fixed), at position i where the observed nucleotide is k while the reference nucleotide is j. P_{ijk} is the true frequency (provided by the user *-mf*) of the corresponding cases similar to O_{ijk}.

To check whether or not the variation within Table 11 is tolerable, we define a null hypothesis (H_0) that the observed mismatches at position 1 in the 5' termini follow the mismatch frequency provided by the misincorporation file (*-mf*) and test if the difference between the observed and expected values are within its expected 95% confidence interval (CI), approximately the difference obeys a normal distribution.

$$\frac{O_{ijk}}{N_{ij}} - \frac{E_{ijk}}{N_{ij}} \sim \mathcal{N}\left(0, \frac{E_{ijk}(1 - E_{ijk})}{N_{ij}}\right) \tag{8}$$

For all nucleotide substitutions for position 1 as shown in Table 10 we find that the difference between the expected and observed mismatch frequency are all within the 95% CI. Hence, H_0 cannot be rejected, which supports that the relative errors in Table 11 are tolerable and that the mismatches are accurately incorporated into the fragment sequences.

4.5 Biological variation model

As described in section 2.2.6, NGSNGS can simulate several genetic variations, including Single-Nucleotide Polymorphisms (SNPs) and structural variations such as insertions and deletions using two models.

The first model incorporates the variations provided by a Variant Calling Format file [11], representing true biological genetic variations for an individual. These variations are incorporated into the original reference genome sequence (*-i*).

We found that NGSNGS simulated sequence reads with successfully incorporated SNPs, insertions and deletions for individuals with different genotypes and ploidy. In the following paragraphs the results for some cases of variations are used to illustrate the validity of the *-vcf* option.

We generated multiple cases of three individuals with one specific type of variation (completely synthetic), either SNPs, deletions and insertions from a haploid or diploid organism. In each case the genotypes of the three individuals are homozygous for the reference, the alternative, or heterozygous.

The variant calling formats illustrated in the following sections only show the variations and not the header information, however the file provided to the *-vcf* requires the header information to be present. To accurately represent an entire population, the vcf file might contain the identified genotypes for several individuals, for which NGSNGS is currently limited to simulate the variations from a single individual, which can be chosen using the *-id* option (zero-indexed). The vcf files contain mitochondrial variations, despite it being a haploid chromosome, we also created a diploid scenario based on the mitochondrial genome.

Our approach to verify the correct incorporation of the provided genetic variations from the vcf file was to compare the original reference genome to the altered reference genome. Using the option *-DumpVCF*. This will write the full internal representation of all paternal chromosomes in a fastafile.

Examining the nucleotide sequence of both the fasta files, of a given positions reveals whether or not the internal fasta files accurately represents the incorporated variations.

4.5.1 Genetic variation - SNP

The vcf file containing the SNP variations represents a haploid genome, with the genotype information of 1 or 0 signifying that the haplotype of that individual for a given position are the alternative allele or the reference allele.

#CHROM	POS	REF	ALT	QUAL	FORMAT	Indiv_0	Indiv_1	Indiv_2
MT	4000	A	T	100	GT	1	0	0
MT	5000	A	C	100	GT	1	0	1
MT	9000	A	T	100	GT	1	0	0
MT	14000	T	A	100	GT	1	0	0
MT	16000	G	A	100	GT	1	0	1

In the diploid representation, "*Indiv_0* and *Indiv_1*" remain homozygous and individual "*Indiv_1*" are converted into heterozygous for all positions.

To simulate the variations for the first individuals from the example above, the user can provide (*-id 0*) with the expectation of solely simulating the alternative allele.

```
./ngsngs -i hs37d5_MT.fa -r 10000 -t 1 -s 100 -l 150 -seq SE -ne -vcf
  ChrMtSNPHaploid.vcf -id 0 -q1 Test_Examples/AccFreqL150R1.txt -chr MT -
  DumpVCF SNPHaID0 -f fq -o MtSNP
```

For diploid genome simulations the option *-DumpVCF* will generate two entries within the internal fasta file representing both parental haplotypes.

Table 12 shows two nucleotides before and after the position of the SNP variation for both the reference genome sequence and the internal fasta file for all three individuals across the ploidy levels.

Reference	>MT:3998-4002	>MT:4998-5002	>MT:8998-9002	>MT:13998-14002	>MT:15998-16002
	TTATT	AAAAT	GTACG	CCTAA	AAGAT
ID 0 GT 1	TTTTT	AACAT	GTTCG	CCAAA	AAAAT
ID1 GT 0	-	-	-	-	-
ID 2 GT 0 or 1	-	AACAT	-	-	AAAAT
ID 0 GT 1/1	TTTTT/<br>TTTTT	AACAT/<br>AACAT	GTTCG/<br>GTTCG	CCAAA/<br>CCAAA	AAAAT/<br>AAAAT
ID 1 GT 0/0	-/-	-/-	-/-	-/-	-/-
ID 2 GT 0/1	-/<br>TTTTT	-/<br>AACAT	-/<br>GTTCG	-/<br>CCAAA	-/<br>AAAAT

Table 12: The extracted sequence of the reference genome, represented in the first row. With the following three rows representing the sequences of the haploid representations, and the last three rows the diploid individuals. Only those sequences distinguishable from the reference genome are illustrated, whereas those sequences identical to the reference genome are represented by a "-".

Table 12 shows that all individuals have been accurately simulated with their expected variation.

Lastly, we also analyse the simulated sequencing reads using the usual genotyping approach, by aligning the the generated fastq files before performing genotype calling. The called SNP variations, the position and the individual genotypes exactly matched the input vcf file, with only the haploid vcf files shown.

#CHROM	POS	REF	ALT	QUAL	FORMAT	Indiv_0	Indiv_1	Indiv_2
MT	4000	A	T	213	GT	1	0	0
MT	5000	A	C	474	GT	1	0	1
MT	9000	A	T	219	GT	1	0	0
MT	14000	T	A	218	GT	1	0	0
MT	16000	G	A	474	GT	1	0	1

Thus, given a vcf comprised of SNPs, NGSNGS is able to accurately simulate the variations for specific individuals for different ploidy levels.

4.5.2 Structural variation - Insertions

We performed a similar analysis as done in section 4.5.1 to identify the insertions using the following vcf file.

#CHROM	POS	REF	ALT	QUAL	FORMAT	Indiv_0	Indiv_1	Indiv_2
MT	1000	T	TTGGA	100	GT	1	0	1
MT	2000	C	CGA	100	GT	1	0	0
MT	6000	C	CGGTTA	100	GT	1	0	0
MT	7000	T	TG	100	GT	1	0	1

As detailed in the VCF specifications [11], the alternative allele includes both the indel sequence and the nucleotide preceding the indel event. As a consequence, the alternative allele at position 1000 which consist of "TTGGA" is in reality the sequence "TGGA" inserted after the reference allele "T" at the given position.

To verify the actual insertion sequence within the internal fasta file, we compare the sequence from the first individual which always simulates the alternative allele to that of the reference.

Reference	MT:998-1002	MT:1998-2002	MT:5998-6002	MT:6998-7002
	ACTCC	TACCG	CTCTA	CGTAC
ID 0 GT 1	MT:998-1006	MT:2002-2008	MT:6004-6013	MT:7009-7014
	ACTTGGACC	TACGACG	CTCGGTTATA	CGTGAC

Table 13: The extracted sequence of the reference genome, represented in the first row, with the following row representing the haploid sequence of the first individual, from the internal *-DumpVCF* fasta file, containing only the alternative insertions. The marked nucleotides represent the entire sequence present in the alternative column in the vcf file.

When performing genotype calling with bcftools we obtained an output vcf file identical to the vcf that was used as an input, confirming accurate simulations of insertions.

#CHROM	POS	REF	ALT	QUAL	FORMAT	Indiv_0	Indiv_1	Indiv_2
MT	1000	T	TTGGA	474	GT	1	0	1
MT	2000	C	CGA	219	GT	1	0	0
MT	6000	C	CGGTTA	219	GT	1	0	0
MT	7000	T	TG	408	GT	1	0	1

4.5.3 Structural variation - Deletions

We use the following vcf file as template for validating the proper inference of deletions.

#CHROM	POS	REF	ALT	QUAL	FORMAT	Indiv_0	Indiv_1	Indiv_2
MT	1000	TCCAGT	T	100	GT	1	0	1
MT	2000	CCGAGC	C	100	GT	1	0	0
MT	6000	CTA	C	100	GT	1	0	0
MT	7000	TACTACACG	T	100	GT	1	0	1

We are able to successfully identify the correct deleted structural variations (highlighted in Table 14) when comparing to the internal fasta file (*-DumpVCF*) but we also see these variations

when we perform proper downstream genotype calling (see Table 15).

Reference	MT:998-1007<br>ACTCCAGTTG	MT:1998-2007<br>TACCGAGCCT	MT:5998-6004<br>CTCTAAG	MT:6998-7010<br>CGTACTACACGAC
ID 0 GT 1	MT:998-1007<br>ACTTGACACA	MT:1993-2002<br>TACCTGGTGA	MT:5988-5994<br>CTCAGCC	MT:6986-6998<br>CGTACACGTACTA

Table 14: The extracted sequence of the reference genome, represented in the first row, with the following row representing the internal haploid sequence of the first individual containing only the alternative deletion. The marked nucleotides represent the entire sequence present in the alternative column in the vcf file.

#CHROM	POS	REF	ALT	QUAL	FORMAT	Indiv_0	Indiv_1	Indiv_2
MT	999	CTCCAGT	CT	228	GT	1	0	1
MT	1999	ACCGAGCC	ACC	228	GT	1	0	0
MT	6000	CTA	C	228	GT	1	0	0
MT	7000	TACTACACGAC	TAC	288	GT	1	0	1

Table 15: Output vcf file obtained using bcftools genotype calling, notice that we infer exactly the same variations as our input vcf file. Some of the variants are shifted with one basepair but then includes an additional nucleotide. This is allowed by the vcf specification and is an artifact of the chosen genotype-calling approach.

4.5.4 Genetic variation - Deamination and Coverage

After verifying the simulations of the genetic variations in sections 4.5.1, 4.5.2 and 4.5.3 we investigated the effect of varying depth of coverage (-c) in our ability to infer variations in combination with post-mortem damage. In the previous section, we used a small chromosome, i.e. the human mitochondrial genome with a size of 16569 base pairs, for this test we used the human chromosome 22, with a length of 51304566. This analysis used a vcf file containing 16583 genetic variations scattered across the entire chromosome. These chosen SNPs are a subset selected from a single diploid individual which is homozygous for the alternative allele on all positions. In Table 16 we observe how many SNPs were successfully identified conditional on the depth of coverage (-c). In this table, we have stratified the analyses into two datasets. The first five rows contain the raw data without including any sequencing error, deamination or other kinds of nucleotide alterations. In the last five rows, we include post-mortem damage. Notice that we obtain nearly identical results which could be indicative of the robustness of the genotype calling method.

Depth of coverage (-c)	0.05	0.1	0.5	1	1.5	2	2.5	5	10
Calculated breadth of coverage (%)	4.745	9.1740	35.052	52.061	60.297	64.295	66.214	67.964	68.014
Number of reads	35968	71935	359717	719413	1079147	1438854	1798581	3597113	7194366
Number of identified variations from VCF	854	1710	6778	10821	13216	14479	15220	16238	16447
Number of non-identified variations from VCF	15729	14873	9805	5762	3367	2104	1363	345	136
Percentage of identified variations (%)	5.150	10.312	40.873	65.254	79.696	87.312	91.7807	97.920	99.180
Calculated breadth of coverage (%)	4.746	9.140	34.986	51.973	60.228	64.242	66.190	67.965	68.014
Number of reads	35967	71932	359720	719399	1079091	1438769	1798468	3596962	7193934
Number of identified variations from VCF	889	1659	6928	10762	13029	14348	15151	16239	16432
Number of non-identified variations from VCF	15694	14924	9655	5821	3354	2235	1432	344	151
Percentage of identified variations (%)	5.361	10.004	41.778	64.898	78.568	86.522	91.365	97.926	99.089

Table 16: The number of identified variations given a specific coverage. Compared to the the input vcf file *-vcf* with a total of 16583 variations. The first 5 rows of information are for simulations without any further sequencing alterations, whereas the last 5 rows are the identified variations when simulating data with deamination added using the Biotin deamination model, as described in section 2.2.4.

From Table 16 we observe that despite the number of reads increasing when the depth of coverage increases from 1.5X to 10X, the actual breadth of coverage increases with a slower rate and seems to stagnate when reaching a value between 66 to 68 % of the chromosome covered. As chromosome 22 contains 32% Ns (due to unmappable regions), this corresponds to close to 100 % breadth of coverage. We do observe a high percentage of correctly identified genetic variations, with 87.312 % correctly identified with a depth of coverage of 2X up to 99.180 % identified for 10X. For a low percentage of identified variants, it doesn't mean the remaining percentages are wrong, but the simulated reads haven't covered those positions making these variations absent from the simulated dataset, as visualized in Figure 17.

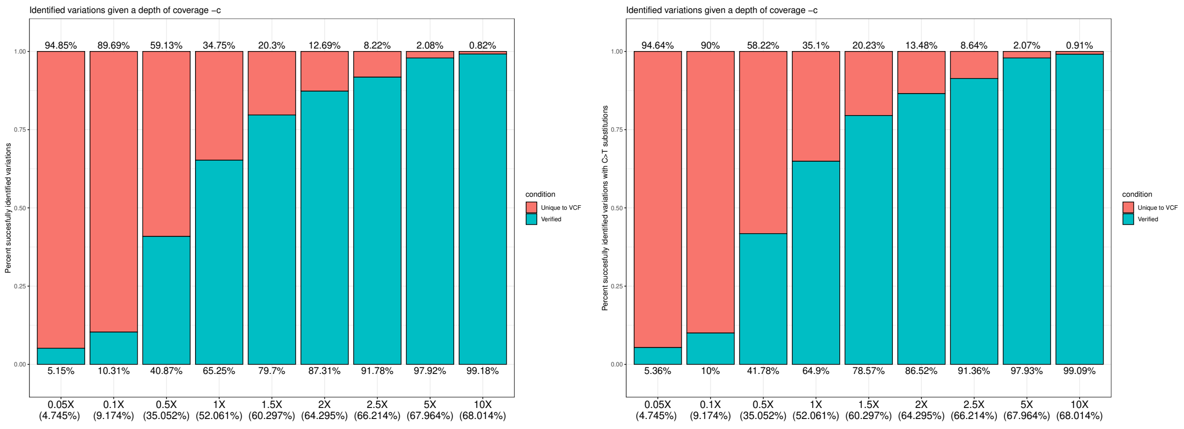

(a) Simulations without deamination.

(b) Simulations with deamination.

Figure 17: Percentage of successfully identified variations from two different runs of simulations, with the varying depth of coverage and the measured breadth of coverage noted below.

From Figure 17a and 17b, we observe a similar pattern between datasets with and without post-mortem damage. The reason being that deamination patterns are specific to the single fragments, whereas the variations are incorporated into the reference genome used as templates to extract all fragments. Therefore the chance of observing the same variation across several reads from the same genomic region, given a high depth of coverage, is higher than observing the

same deamination pattern. This accounts for the similar pattern of percent successfully identified variations (Figure 17) across the simulations with- and without deamination. Interestingly we do identify more variations when using the *-vcf* option along with the deamination simulations, all of which are SNP variations in the form of PMD as the reference and alternative alleles being C and T or G and A.

4.6 Stochastic variation model

When simulating indels (*-indel InsProb,DelProb,InsParam,DelParam*) the user can specify the probability of having either insertions or deletions (*InsProb*, *DelProb*). The lengths of the insertions or deletions are modeled by a geometric distribution with a given parameter ranging from 0 to 1 (*InsParam*, *DelParam*).

$$P(Y = k) = p(1 - p)^{k-1}, \quad k = 1, 2, 3, 4... \tag{9}$$

where p represents either *InsParam* or *DelParam*.

Contrary to the real biological variants that form true changes to the haplotypes of the sample, the stochastic indels are simulated at the fragment level and it is therefore not meaningful to verify at the genotype calling level.

Similar to the previously described output option (*-DumpVCF*) we offer the user the possibility of keeping track of the simulated stochastic indels (*-DumpIndel*, example in Table 17).

```
./ngsngs -i Test_Examples/Mycobacterium_leprae.fa.gz -r 1000 -t 1 -s 1 -lf
    Test_Examples/Size_dist_sampling.txt -seq SE -ne -indel 0.0,0.05,0.0,0.9 -q1
     Test_Examples/AccFreqL150R1.txt -DumpIndel IndelPos -f fq -o IndelOut
```

Table 17 shows two examples of entries from the internal *-DumpIndel* information files recording only insertions and only deletions from two separate simulation runs.

Read ID	Number of insertions	Insertions operations	Number of deletions	Deletion operations	Read length before indel	Read length after indel
T0_RID227_S0_NZ_CP029543.1:325855-325896_length:42_mod0010	1	[19,19:2]	0	-	42	44
T0_RID70_S0_NZ_CP029543.1:1884007-1884048_length:42_mod0010	2	[7,7:1],[33,34:3]	0	-	42	46
T0_RID34_S0_NZ_CP029543.1:2565205-2565244_length:40_mod0020	0	-	1	[37,37:2]	40	38
T0_RID645_S0_NZ_CP029543.1:98920-98959_length:40_mod0020	0	-	2	[5,5:1],[22,21:1]	40	38

Table 17: The first two rows illustrates the read id with 1 and 2 insertions. The final two rows illustrates the read id of sequences with 1 and 2 deletions.

In table 17 the third and fifth column stores information regarding the position (zero-indexed) and number of affected nucleotides by the insertion and deletion operations respectively, structured as [*position in original fragment*, *position in altered fragment*:*length of indel*]. For the first indel operation, the position within the original and altered fragment remains identical, whereas they diverge for all of the following indel occurrences, with the position within the altered fragment increasing compared to the original fragment for insertions (second row, third column, second operation [*33*,*34*:*3*]), and decreases for deletions (fourth row, fifth column, second operation [*22*,*21*:*1*]).

Besides the recorded positions, comparing the actual sequence reads to the respective reference genome region (which can be identified within the read ID section 2.4), can serve to further support accurate simulations.

The simulated sequencing reads with insertions from Table 17 and the corresponding reference genome sequence, shows sequence insertions (red highlights) at the correct positions and length (surrounding nucleotides blue highlight).

```
>NZ_CP029543.1:325855-325896
TGCACGTGCTGATTGCTCATTCGCTGGCCAAGGGGCCCGGGT
@T0_RID227_S0_NZ_CP029543.1:325855-325896_length:42_mod0010 R1
TGCACGTGCTGATTGCTCANTTTCGCTGGCCAAGGGGCCCGGGT
>NZ_CP029543.1:1884007-1884048
CTAGTGCCATACTTGACCGGATAAAACACCGGATAAAACCTT
@T0_RID70_S0_NZ_CP029543.1:1884007-1884048_length:42_mod0010 R1
CTAGTGCACATACTTGACCGGATAAAACACCGGATCCTAAAACCTT
```

The simulated sequencing reads, likewise showed correct placement and lengths of the deletions (Table 17).

```
>NZ_CP029543.1:2565205-2565244
ACGGGCTACCTTCGCTGGATCGCCGCACTGGAATGTGACC
@T0_RID34_S0_NZ_CP029543.1:2565205-2565244_length:40_mod0020 R1
ACGGGCTACCTTCGCTGGATCGCCGCACTGGAATGTGC
>NZ_CP029543.1:98920-98959
TCGATCGCCGCCAGATAGATATCGGTTAGCCGGGTGCACG
@T0_RID645_S0_NZ_CP029543.1:98920-98959_length:40_mod0020 R1
TCGATGCCGCCAGATAGATATGGTTAGCCGGGTGCACG
```

Thus, we can conclude that NGSNGS reliably simulates indels using this stochastic variation model.

References

[1] J. K. Bonfield, J. Marshall, P. Danecek, H. Li, V. Ohan, A. Whitwham, T. Keane, and R. M. Davies, "Htslib: C library for reading/writing high-throughput sequencing data," *Gigascience*, vol. 10, no. 2, p. giab007, 2021.

[2] H. Li, B. Handsaker, A. Wysoker, T. Fennell, J. Ruan, N. Homer, G. Marth, G. Abecasis, and R. Durbin, "The sequence alignment/map format and samtools," *Bioinformatics*, vol. 25, no. 16, pp. 2078–2079, 2009.

[3] Q. Fu, H. Li, P. Moorjani, F. Jay, S. M. Slepchenko, A. A. Bondarev, P. L. Johnson, A. Aximu-Petri, K. Prüfer, C. De Filippo, *et al.*, "Genome sequence of a 45,000-year-old modern human from western siberia," *Nature*, vol. 514, no. 7523, pp. 445–449, 2014.

[4] Y.-C. Chen, T. Liu, C.-H. Yu, T.-Y. Chiang, and C.-C. Hwang, "Effects of gc bias in next-generation-sequencing data on de novo genome assembly," *PloS one*, vol. 8, no. 4, p. e62856, 2013.

[5] A. J. Walker, "New fast method for generating discrete random numbers with arbitrary frequency distributions," *Electronics Letters*, vol. 10, no. 8, pp. 127–128, 1974.

[6] W. Huang, L. Li, J. R. Myers, and G. T. Marth, "Art: a next-generation sequencing read simulator," *Bioinformatics*, vol. 28, no. 4, pp. 593–594, 2012.

[7] A. W. Briggs, U. Stenzel, P. L. Johnson, R. E. Green, J. Kelso, K. Prüfer, M. Meyer, J. Krause, M. T. Ronan, M. Lachmann, *et al.*, "Patterns of damage in genomic dna sequences from a neandertal," *Proceedings of the National Academy of Sciences*, vol. 104, no. 37, pp. 14616–14621, 2007.

[8] M. Meyer and M. Kircher, "Illumina sequencing library preparation for highly multiplexed target capture and sequencing," *Cold Spring Harbor Protocols*, vol. 2010, no. 6, pp. pdb–prot5448, 2010.

[9] G. Renaud, K. Hanghøj, E. Willerslev, and L. Orlando, "gargammel: a sequence simulator for ancient dna," *Bioinformatics*, vol. 33, no. 4, pp. 577–579, 2017.

[10] H. Jónsson, A. Ginolhac, M. Schubert, P. L. Johnson, and L. Orlando, "mapdamage2. 0: fast approximate bayesian estimates of ancient dna damage parameters," *Bioinformatics*, vol. 29, no. 13, pp. 1682–1684, 2013.

[11] P. Danecek, A. Auton, G. Abecasis, C. A. Albers, E. Banks, M. A. DePristo, R. E. Handsaker, G. Lunter, G. T. Marth, S. T. Sherry, *et al.*, "The variant call format and vcftools," *Bioinformatics*, vol. 27, no. 15, pp. 2156–2158, 2011.
